# A randomized controlled trial investigating digital resilience training for healthcare professionals

**DOI:** 10.1038/s41598-025-28028-z

**Published:** 2025-12-24

**Authors:** Ying Lau, Kai Chow Choi, Sai Ho Wong, Wen Wei Ang, Wei How Darryl Ang, Siew Tiang Lau

**Affiliations:** 1 The Nethersole School of Nursing, The Chinese University of Hong Kong, Hong Kong Special Administrative Region, Sha Tin, Hong Kong, China; 2 Alexandra Hospital, Singapore, Singapore; 3 Alice Lee Centre for Nursing Studies, Yong Loo Lin School of Medicine, National University of Singapore, Singapore, Singapore

**Keywords:** Healthcare professionals, Digital resilience training, Work engagement, Intention to leave, Counterproductive work behavior, Psychology, Health care, Health occupations

## Abstract

The unpredictable coronavirus disease and complexity of healthcare settings have caused emotional exhaustion and burnout among healthcare professionals globally. Building resilience at work training can facilitate a change in healthcare professionals’ overall ability to bounce back from adversity. The study aimed to develop, validate, and evaluate a digital resilience training (BRAW) for healthcare professionals in Singapore. Considering the multifactorial nature of resilience at work, the content of the 6-session resilience training was developed based on systematic reviews and psychological theories. The overall rating was 85% of the total points across five experts using the Conduct and Health-Related Website Evaluation Form, indicating valid content. A two-armed randomized controlled trial was used among 410 healthcare professionals. Primary outcome was resilience, and secondary outcomes included work engagement, intention to leave, and counterproductive work behavior. Three study time points were assessed. A generalized estimating equations model showed that the healthcare professionals in the BRAW had significant improvement in resilience, at the post-intervention and/or 3-month follow-up. Findings suggested that BRAW can be considered as supplementary training for healthcare professionals to equip themselves for handling unpredictable pandemics in the future. Further research is needed to examine the long-term effects and generalizability of digital resilience training.

**Clinical trial numbers**: (ClinicalTrials.gov Identifier: NCT05130879, first registration date: 23/11/2021)

**Supplementary Information:**

The online version contains supplementary material available at 10.1038/s41598-025-28028-z.

## Introduction

Working in healthcare has been known to be arduous and grueling^1^. The healthcare profession is challenging due to its shift work, physical workload, and high-strung nature, which can lead to high stress, emotional exhaustion, fatigue, and burnout, ultimately leading to sickness absence and high turnover^2^. The global prevalence of low resilience was 26% (95% CI: 20–32) in healthcare professionals across 16 countries^3^. The current working environment is turbulent and complex owing to increasing globalization and digitalization^4^. The ongoing change in healthcare setting increases pressure and challenges for healthcare professionals^5^. Following the initial reports of coronavirus disease in 2019 (COVID-19), the virus-related respiratory illness has proliferated internationally, including Singapore, and has caused a global pandemic^6^. Healthcare professionals are crucial to the workforce in response to the ongoing COVID-19 pandemic to maintain patient care^7^. According to systematic reviews^8,9^, 28% of healthcare personnel reported feeling unfulfilled personally, 51% reported feeling emotionally exhausted, 52% reported feeling depersonalized, and 14.7% to 76% reported experiencing burnout symptoms during the COVID-19 pandemic. The study recommends training for all healthcare professionals to help them better cope with the devastating effects of the pandemic and build resilience for future unpredictable pandemics^10^.

Resilience, one of the core constructs of positive organizational behavior, may play a significant role in explaining the ability to survive and thrive in the face of workplace adversity^11^. Resilience is the ability to build strengths and virtues for sustainable high performance and well-being based on experiencing and coping with adverse events^12^. Resilience can be both an outcome and a dynamic process encompassing positive adaptation in significant adversity^13^. From a process perspective, both resilience mechanisms and resilience-promoting factors may influence resilience outcomes^11^. Healthcare professionals reacted and behaved in the face of adversity using their coping strategies or emotional responses via resilience mechanisms^13^. Resilience-promoting factors refer to personal or environmental characteristics that can buffer the negative effects of adversity or enhance resilience mechanisms during adverse experiences^13^. Becoming resilient healthcare professionals helped to deal with the challenges faced at work. Hence, building resilience at work is necessary because adversity is unavoidable for healthcare professionals.

Resilience training has led to adaptive changes in resilience-promoting factors, enhancing healthcare professionals’ ability to adapt effectively during challenging circumstances over time^14,15^. Indeed, a growing body of research suggests resilience training at work may play a pivotal role in the realm of prevention, particularly concerning protecting the long-term well-being of healthcare professionals^14,16^. Resilience training aims to develop the capacity of healthcare professionals to bounce back and protect their well-being and performance in the face of adversity^17^. Considering the multifactorial nature of resilience at work, our study team utilized essential concepts from the following three theories to develop the content of resilience training.

Cognitive behavior therapy (CBT) is a form of psychotherapy that assumes that all psychological disturbances are caused by dysfunctional thinking^18^. It aims to modify thoughts, beliefs, and perceptions and change behavioral patterns^18^. When confronted with stress or adversity, healthcare professionals show maladaptive behavioral responses or experience negative mood states, or both, due to irrational cognitions. This leads to the transformation of cognitive processes into more flexible emotional and behavioral reactions to stress. It might contribute to the enhancement of psychological resilience in healthcare professionals^14^.

Psychopathology is essentially the sequence of psychological inflexibility (i.e., the inability to persist or change behavior according to long-term values due to cognitive skills), according to acceptance and commitment therapy (ACT)^19^. By learning acceptance and mindfulness skills (such as being in contact with the present moment, acceptance and cognitive diffusion) and commitment and behavior-change skills (such as values and committed action)^19^, several resilience-promoting factors might be enhanced. Hence, ACP might foster cognitive flexibility and purpose in life that might result in a better adjustment to stressful working conditions^19^.

Resilience training focuses on a healthcare professional’s capacity to manage stressful situations and adverse circumstances in the workplace more effectively and with greater emotional insight^16^. Effective problem-solving therapy (PST) can attenuate the negative effects of stress and adversity on well-being by moderating and mediating the effects of stressors^20^. Resilience training based on PST enhances healthcare professionals’ positive problem orientation. Healthcare professionals can analyze the problem, generate possible solutions, choose the best solution, create an action plan, implement the solution, and review the problem-solving process. These processes may foster adaptation to stress by improving the resilience-promoting factors of active coping^20^.

Most of the resilience training involving multiple face-to-face sessions^21,22^. Although the face-to-face delivery format was effective^21,22^, time and intensive resources are needed with limited accessibility. However, this is a particular challenge for many healthcare professionals, where taking healthcare professionals away from the workplace to attend training creates considerable disruption to the workforce in clinical settings. In addition, the associate costs for replacing healthcare professionals during this time can be significant. The expense inherent in face-to-face training can pose a hindrance, as can the availability of trainers and training in remote areas. Moreover, stigma associated with psychological problems remains prevalent and may prevent a subset of healthcare professionals from high attrition rate. Hence, digital resilience training with universal preventive approach is a potential solution.

Over the recent decades, digital interventions are advancing at an unprecedented pace and have been integrated into resilience training in workplace^23–25^. The internet can reach individuals worldwide and enable interaction without geographic boundaries. A meta-analysis highlighted online resilience training has make the training relatively easy and cost-effective on a large scale^22^. The internet makes training feasible and worth considering. Digital health technology creates new opportunities to enhance functionality, connectivity, and manageability.

There are several systematic reviews on digital interventions^26,27^ or workplace resilience training^16,21,22^. For two digital interventions, one used digital resilience training^27^ and the other used web-based psychological intervention^26^. The meta-analytic results showed that digital intervention had moderate to large effect in enhancing resilience^27^ and small effect in improving psychological well-being and work effectiveness^26^. However, overall quality of selected RCTs in two reviews was low due to selection and reporting biases^26,27^. For workplace resilience training, one review employed a meta-analysis^22^, while two others utilized narrative syntheses^16,21^. Three reviews did not evaluate the quality of the studies. A meta-analytic review revealed that there was a small effect of the resilience-building program at work on performance and well-being outcomes^22^. Narrative analyses indicated that workplace resilience interventions benefited healthcare professionals and employees by enhancing resilience, well-being, engagement, and mental health^16,21^. Given that previous reviews and meta-analyses identified poor-quality RCTs on resilience training in the workplace, there is a need for future well-designed RCTs.

Digital health technology can facilitate the delivery of resilience training^28^. The workplace has integrated internet-based interventions, which have advanced at an unprecedented pace in recent decades, into resilience training. We found that three primary studies were conducted involving 93 nurses in the United Kingdom^29^, 83 healthcare professionals in Italy^30^, and 148 healthcare professionals in the United States^31^. One study found that there was no significant difference in resilience between the training group and the control group^29^, while another study showed that the training group had a significant change in resilience over time^31^. A single group pre-post study revealed significantly improved resilience after training^30^. However, one study used pilot randomized controlled trial^29^ and two studies used a non-randomized research design^30,31^. Despite the insights provided by previous studies on resilience training in the workplace, the effectiveness of such training for healthcare professionals in Singapore remains uncertain.

Resilience training may be effective on objective business measures such as intention to leave, work engagement, employability, and work performance, but there is a lack of literature on these outcomes. We found two RCTs to evaluate a smartphone-based stress management program that can improve work engagement among nurses in Vietnam^32^ and a digital intervention that cannot improve the intention to leave among newly licensed graduate nurses in the United States^33^. Resilience training may influence healthcare professionals’ turnover intentions both directly^34^ and indirectly^35^ in two observational studies. Two quasi-experimental studies showed that healthcare professionals in job-crafting interventions were associated with job performance^36^. As a result, there was insufficient direct evidence of resilience training in terms of intention to leave, work engagement, employability, and work performance. To fill the research gap, the objectives of this research were to develop, validate, and evaluate the effectiveness of BRAW training on resilience, work engagement, intention to leave, employability, and work performance.

## Methods

### Development of evidence-driven digital resilience intervention

Our research team developed a digital resilience intervention for healthcare professionals in Singapore, and we named it “Building Resilience at Work (BRAW).” To design of essential features of the digital resilience program, our research team based on two systematic reviews^16,27^. One review focused on digital resilience training, which was based on 21 RCTs across 11 countries. Another systematic review focused on workplace intervention, drawing from 33 studies conducted in 10 countries^16^. Most interventions used ranged from 4 to 8 sessions, and 6 sessions were the most preferable number of sessions. The subgroup analysis revealed that the internet with a flexible program schedule was more effective for building resilience^27^. However, we observed a small sample size with low quality in existing trials, indicating the need for a future well-designed trial.

The program aimed to enhance resilience, work engagement, intention to leave, employability, and work performance among healthcare professionals. The BRAW involves a range of skills and strategies drawn from evidence-based therapies, including CBT^18^, ACT^19^, and PST^20^. We tailored the contents to healthcare professionals based on recommended resilience programs from systematic reviews^16,27^. Tables S1 and S2 display the BRAW’s content and screenshots. The training incorporates online sessions, self-monitoring, homework and exercise, peer support, therapist support, and safety protocol. Each piece of content included two short videos, three short quizzes, a forum, and one to two homework exercises. Each session took about 20–25 min to complete on a smartphone, tablet, laptop, or desktop that connected to the internet. Healthcare professionals can complete the training components at their own pace within a flexible schedule. Participants accessed the six-session training through a password-protected link. For the six sessions delivered every week, we made one session available each week to encourage participants to complete it before moving on to the next. The research team used the built-in rules feature in Microsoft Outlook to automate weekly emails that inform participants about the availability of the next session. If the participants did not access the session, the research team used the short message service did the reminder for them. If participants did not access the session, quiz, or finish the homework, the research team sent them a reminder via short message service.

### Evaluation of the BRAW training

A randomized two-armed parallel control trial was adopted. The design and analysis followed the consolidated standards of reporting trial guidelines (CONSORT) for an internet-based intervention^37^. The trial was registered in the clinical registry (NCT05130879). Table S3 illustrates the CONSORT checklist.

### Sampling and sample size

This study targeted healthcare professionals in Singapore. Based on the previous similar trial^25,38^, we estimated that a total sample of 378 participants (189 per group) would provide 80% power at a 5% level of significance (two-sided) to detect a minimal difference of 1.5 scores on resilience after 6 weeks of BRAW between the intervention and control groups, assuming a standard deviation is 5.2 scores on resilience. We consider a response rate of 72%^25^ and a retention rate of 84%^38^, according to previous studies. A total of 749 samples were invited to participate in the study to achieve the final sample size. This study included healthcare professionals who (1) were 21 years of age or older, (2) were physicians, allied health professionals, medical social workers, case managers, registered nurses (including nurse clinicians, nurse educators, and nurse managers), enrolled nurses, nurse assistants, or patient care assistants, (3) could read English, (4) owned and regularly used smartphones, tablets, laptops, and desktops, and (5) could access the Internet.

We excluded participants with a lifetime history of psychosis, severe depression, personality disorder, or substance abuse at any point in their life. These conditions were excluded because they might pose challenges to the study, increase the risk of adverse outcomes, and impact on the research outcomes related to resilience, work engagement, intention to leave, employability, and work performance^39,40^.

### Recruitment

The research team approached healthcare professionals who had met the sample inclusion criteria from social media platforms such as Facebook, Instagram, LinkedIn, Google Ads, the websites of different healthcare associations, etc., using the QR code of Qualtrics as a sign-in platform. The sign-in platform included the checklist for eligibility criteria, as well as email and mobile contact information. An experienced research assistant contacted eligible participants via mobile phone or Zoom meeting to explain the study’s aims, potential benefits, and risks, and to provide them with the information sheet. The signed consent forms were obtained from all participants before they participated in the study.

### Randomization and allocation

A research team member, who was not involved in the implementation of the study, generated a randomization sequence using a computer-generated algorithm. We randomly assigned them at a 1:1 ratio to one of two conditions after the baseline measurement. We concealed the allocation in consecutively numbered, opaquely sealed envelopes.

### Blinding

We blinded research assistants and participants to the condition until they completed baseline assessments, at which point we opened envelopes to reveal whether they were in the intervention or waitlist condition. We did not explicitly inform the participants about their study condition; however, their participation in the intervention necessitated full blinding.

### Intervention group

Different types of devices connected to the internet, including smartphones, tablet laptops, and desktops, present core content. The program consisted of six online sessions. Based on the allocation number, the research team delivered each session every week to the participant according to the scheduled date. Participants accessed BRAW training for six weeks without a time limit. Participants were reimbursed S$60 (US$46) for the time and effort taken to complete the study and answer the questionnaires in three-time points.

### Control group

The study used a waitlist control group, which served as an untreated comparison group. Participants received BRAW after the 3-month follow-up assessment. We assumed that the waitlist condition exhibited no effects, in line with similar interventional studies.

### Measures

Through an online Qualtrics survey platform, we assessed outcomes using self-rated questionnaires that included demographic and working profile, satisfaction of training, and five validated measures. The primary outcome was resilience, and the secondary outcomes included work engagement, intention to leave, employability, and work performance. Three study time points were used to assess all outcomes at baseline (Time [T] 0), immediate after training (T1), and 3 months after training (T2). We collected demographic and work profiles such as age, race, sex, discipline, professional qualification, marital status, area of work, duration as a healthcare professional, and duration in the institution.

### Resilience

We used a 6-item Brief Resilience Scale (BRS)^41^ to assess the ability to recover from stress. The BRS was a factor scale. We rated the items on a 5-point Likert scale, ranging from 1 (strongly disagree) to 5 (strongly agree). The total score ranged from 6 to 30, with higher scores signifying greater bounce-back resilience. BRS has demonstrated excellent reliability and validity^42^.

### Work engagement

We used a 9-item Utrecht Working Engagement Scale short version (UWES-9)^43^ to measure participants’ feelings in the context of work. The UWES-9 comprised three scales: “vigor,” “dedication,” and “absorption.” Participants rated how often they experienced these feelings on a 4-point Likert scale from 1 (never) to 4 (always). The total score ranged from 9 to 36, and higher scores on the UWES-9 indicate more work engagement. UWES-9 has confirmed satisfactory validity and reliability^44^.

### Intention to leave

We measure the intention to leave using a 12-item Anticipated Turnover Scale (ATS)^45^. The ATS had a unitary structure. The ATS asked questions about the anticipated length of time before leaving and the certainty of leaving the job. We rated the items on a 5-point Likert scale, ranging from 1 (strongly disagree) to 5 (strongly agree). The total score ranged from 12 to 60, with higher scores reflecting a higher degree of turnover. Items had a CVI of 0.80–0.95. Cronbach’s alphas were 0.85–0.94 in several studies^46^. The test-retest reliability coefficient was 0.84 in 2 weeks’ interval^46^.

### Employability

We used an 11-item Self-perceived Employability Scale (SPE)^47^ to assess employability. The SPE included two constructs, namely the “internal employability” and “external employability” subscales. Participants were required to state their agreement with the items by selecting a number on a 5-point scale, from 1 (strong disagreement) to 5 (strong agreement). The total score ranged from 11 to 55, with a higher score indicating perceived superior employability. SPE has reported good internal consistency^47^.

### Work performance

We used an 18-item Individual Work Performance Questionnaire (IWPQ) to measure individual work performance^48–50^. The IWPQ had a recall period of 3 months and a rating scale from 0 (seldom/never) to 4 (always/often) for three subscales, namely “task”, “contextual performance”, and “counterproductive work behavior”. We calculated a mean score by adding the item scores and dividing their sum by the number of items ranging from 0 to 4, with higher scores indicating higher individual work performance. The psychometric properties of the IWPQ indicated excellent internal consistency and good validity^49^.

### Satisfaction with training

We used the self-developed eight-item scale to assess satisfaction with the online training at the end of the six-week training. Items included “Quality of training received”, “Training matched your anticipation”, “Training met your needs”, “Recommend the training to friends”, “Satisfaction with the amount of help received”, “The training received helped you to deal with problems more effectively”, “Overall satisfaction with the training received”, and “If you were to seek help again, would you come back to the training?”. We rated each item on a 4-point Likert scale from 1 to 4, with different response options for each item.

### Data collection

The data collection was conducted from November 15, 2021, to July 27, 2023. At baseline (T0), immediate after training (T1), and 3 months after training (T2), we invited all participants to complete assessments using an online Qualtrics survey platform on their mobile devices. The research assistants demonstrated the steps for exploring BRAW training via a live demonstration on mobile devices. After a week, the participants received a text or email informing them of the next available session. Each participant received a unique user identification, ensuring their confidentiality. To ensure an adequate dosage effect of the intervention, the research team monitored the use of BRAW training, and all participants received weekly reminders. Participants received a list of mental health and crisis management resources for healthcare professionals in Singapore during the first session through an automated email. All participants had access to a question-and-answer platform via the Microsoft Team application, and an experienced research staff, who held a master’s degree in health psychology, provided professional support.

### Statistical analysis

We assessed the normality of continuous variables using their skewness statistics and normal probability plots. We summarized and presented the demographic and work profile characteristics of the participants, along with the outcome measures across study time points, as frequencies (percentages) for categorical variables and as means (standard deviations) for continuous variables. A generalized estimating equations (GEE) model was used to assess the differential changes of each of the outcomes across the study time points (T0: baseline, T1: immediate after training, and T2: 3 months after training) between the two groups^51^. We set a dummy variable (group) to represent the intervention group, using the control group as a reference. We set two dummy variables (T1 and T2) to represent the two time points (T1 and T2), using the baseline (T0) as the reference. We analyzed all outcome variables using the intention-to-treat (ITT) principle, ensuring the prognostic balance from the original random treatment allocation^52^.

We used the interaction terms of the group and time point dummy variables in the GEE models to see how the outcomes changed between the two groups at T1 and T2 compared to T0. The GEE model can handle repeated measures that are correlated with each other and give unbiased estimates using a quasi-likelihood estimation method, even if some data is missing (MCAR)^53^, provided the sample size is not too small and the data are missing randomly. We assessed the plausibility of MCAR using the Little MCAR test. We calculated Hedges’ *g* effect sizes and their associated 95% confidence intervals for the outcomes based on their change scores from T0 to T1 and T2 between the control and intervention groups. We performed all the statistical analyses using IBM SPSS 29.0 (IBM Crop, Armonk, NY). All statistical tests were two-tailed, and a *P*-value < 0.05 was considered statistically significant.

### Ethical considerations

The research team sought ethical approval from the Institutional Review Board with the number NUS-IRB-2021-703. All participants were provided with informed consent during the study. The research assistant gave the participants time to understand the research’s information. The research assistant explained the research to all participants and allowed them to ask questions. The research assistant obtained informed consent once the participants expressed their satisfaction and desire to participate in the study. The participants received a copy of their information sheet. Participation in this study was voluntary. Participants could stop participating in this study at any time.

## Results

### Validation of the BRAW training

Our team designed the BRAW following the eight ethical principles of the Health on the Net Code (HON) of Conduct to ensure the ethical standard for certifying the quality of health-related websites^54^. It included authoritativeness, complementarity, privacy, attribution, justifiability, transparency, financial disclosure, and advertising policy. A panel of five experts, comprising two clinicians, one educationalist, and two educators, evaluated the quality standard of BRAW using the Health-Related Website Evaluation Form (HRWEF)^55^. The nongovernmental organization HON Foundation uses the HRWEF tool in their code of conduct to validate the quality of health-related websites^54^. We used this tool to assess the content, credibility, currency, accuracy, reliability, readability, and design of Internet-based information. To ensure quality, the overall rating of the designed BRAW must meet at least 75% of the total possible points. In our study, the overall rating was 85% of the total possible points across five experts, indicating BRAW training achieved a good standard.

### Effectiveness of the BRAW training

**Fig. 1:**
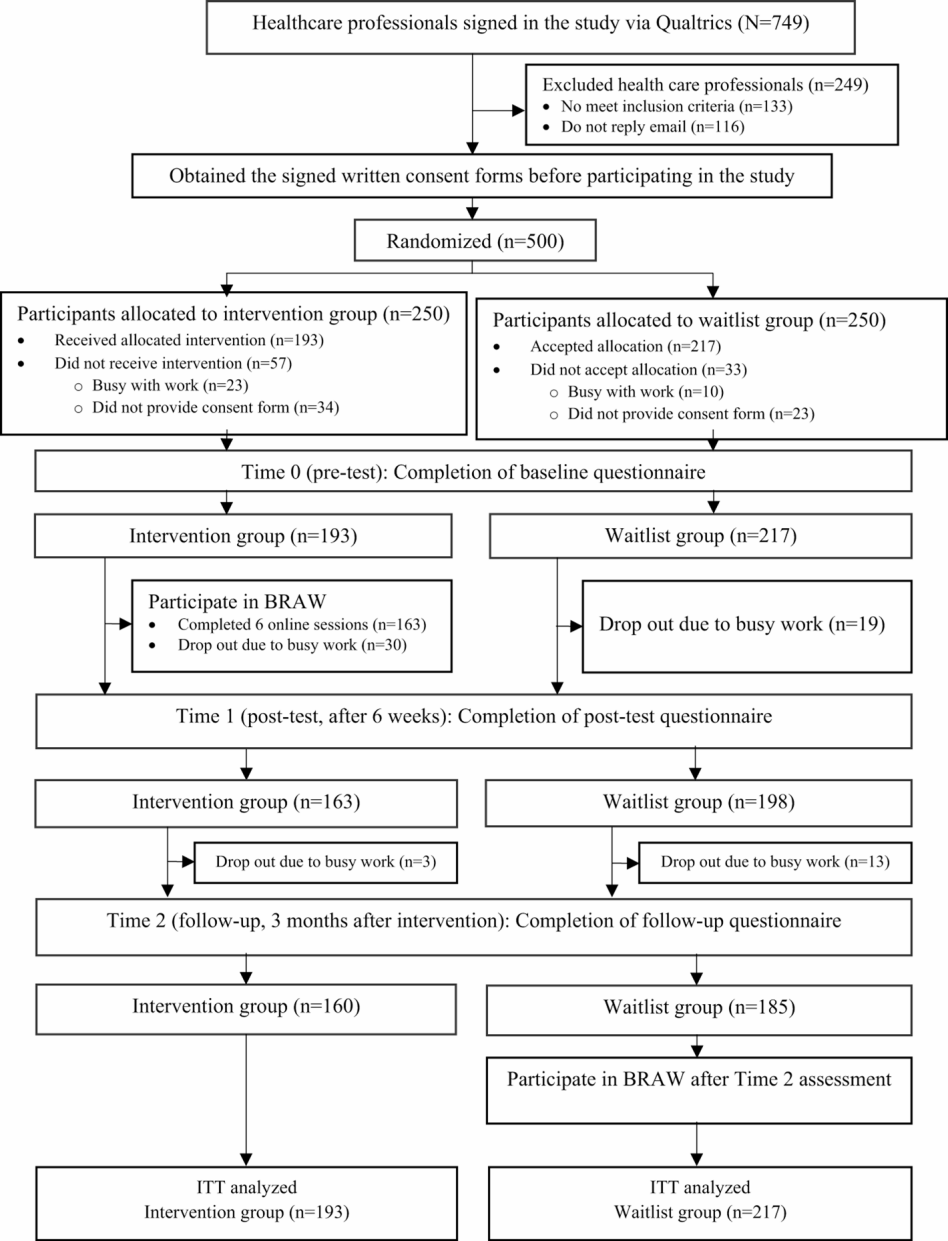
Participant flow diagram.

Figure 1 presents the study flow diagram. Initially, a total of 749 signed in the study via Qualtrics XM. However, we excluded 249 of them because they did not meet the inclusion criteria and did not respond to emails asking them to sign the consent form. The study enrolled 500 eligible healthcare professionals, randomly assigning them to the control (*n* = 250) and intervention (*n* = 250) groups. Before baseline assessments, 90 of them withdrew from the study because they were busy with their work or did not provide consent forms.

The analysis included 217 and 193 participants in the control and intervention groups, respectively, who completed the baseline assessments. Among the 410 participants, 65 (16%) did not complete the outcome assessments at T1 (immediately after training) and/or T2 (3 months after training). The main reason for dropping out was being busy with their work. The baseline characteristics among those who dropped out and those who competed in the study were statistically comparable (all *P* > 0.05, Table S4). The Little MCAR test for the outcome variables was not significant (*P* = 0.296), which supported the plausibility that the data was missing completely at random. No continuous variable was found deviating from the normal distribution.

### Characteristics of participants in randomized controlled trial

Most participants were between 26 and 40 years old (61%), female (83%), and of Chinese ethnicity (83%). All participants worked in hospitals. Most of them had a bachelor’s or higher degree (87%). Nearly half of the participants were nurses (48%). The demographic and work profile characteristics were generally comparable between groups, except that slightly higher proportions of participants were in the youngest age group or had a certificate or diploma professional qualification only in the intervention group when compared with the control group. Table 1 displays the baseline characteristics of the participants in detail.

**Table 1:** Characteristics of healthcare professionals. (*P < 0.05).

Characteristics	All (*N* = 410)		Control (*N* = 217)		Intervention (*N* = 193)		*P*
	*N*	%	*N*	%	*N*	%	
Age (years)							0.01*
≤ 25	96	23.4	40	18.4	56	29.0	
26–30	110	26.8	58	26.7	52	26.9	
31–40	142	34.6	89	41.0	53	27.5	
> 40	62	15.1	30	13.8	32	16.6	
Sex							0.37
Female	339	82.7	176	81.1	163	84.5	
Male	71	17.3	41	18.9	30	15.5	
Marital status							0.86
Married	159	38.8	85	39.2	74	38.3	
Single/divorced/others	251	61.2	132	60.8	119	61.7	
Ethnicity							0.94
Chinese	339	82.7	179	82.5	160	82.9	
Malay	31	7.6	17	7.8	14	7.3	
Indian	32	7.8	16	7.4	16	8.3	
Others	8	2.0	5	2.3	3	1.6	
Profession							0.26
Physician	28	6.8	17	7.8	11	5.7	
Nurse	198	48.3	106	48.8	92	47.7	
Allied health professional	118	28.8	66	30.4	52	26.9	
Others	66	16.1	28	12.9	38	19.7	
Professional qualification							0.01*
Certificate/Diploma	54	13.2	23	10.6	31	16.1	
Bachelor’s degree	275	67.1	140	64.5	135	69.9	
Master’s degree or higher	81	19.8	54	24.9	27	14.0	
Area of work							0.99
Hospital	410	100.0	217	100.0	193	100.0	
Years of experience as health care worker							0.60
< 1	59	14.4	30	13.8	29	15.0	
1–2	49	12.0	24	11.1	25	13.0	
> 2–5	92	22.4	46	21.2	46	23.8	
> 5–10	81	19.8	44	20.3	37	19.2	
> 10–20	96	23.4	58	26.7	38	19.7	
> 20	33	8.0	15	6.9	18	9.3	
Years in institution							0.95
< 1	80	19.5	45	20.7	35	18.1	
1–2	69	16.8	35	16.1	34	17.6	
> 2–5	111	27.1	58	26.7	53	27.5	
> 5–10	75	18.3	41	18.9	34	17.6	
> 10–20	59	14.4	31	14.3	28	14.5	
> 20	16	3.9	7	3.2	9	4.7	

### Outcome measures across the study time points

Table 2 presents descriptive statistics of all outcomes at the three study time points. The Hedges’ *g* effect sizes of the outcomes based on the standardized mean difference of their change scores at T1 and T2 with respect to T0 are also provided in Table 2. Table 2 shows the descriptive statistics (means and standard deviations) of the outcome scores across the study time points between the control and intervention groups as well as estimated effect sizes of the intervention on the study outcomes. A widely adopted guideline for interpretation^56^ states that effect sizes of magnitudes 0.2, 0.5 and 0.8 can be interpreted as small, medium, and large, respectively. By this standard, the intervention might have a small to medium effect on resilience but only small effects on work performance and work engagement.

**Table 2:** Outcome measures across study time points between the two groups. (BRS = Brief resilience scale; UWES-9 = The 9-item Utrecht working engagement scale short version; ATS-12 = The 12-item anticipated turnover scale; SPE = Self-perceived employability scale; IWPQ = Individual work performance Questionnaire).

Outcomes		Mean (SD) for each time point		Hedges’ g effect size (control vs. intervention) (95% CI)
		Control	Intervention	
Resilience				
BRS Total score	T0	18.7 (3.9)	18.3 (4.2)	
[Possible score range: 6–30]	T1	19.4 (3.9)	20.1 (4.1)	0.38 (0.18, 0.57)
	T2	19.4 (4.2)	20.8 (3.8)	0.51 (0.32, 0.71)
Work engagement				
UWES-9 Vigour subscale score	T0	9.9 (3.3)	10.0 (3.4)	
[Possible score range: 0–18]	T1	10.1 (2.9)	10.7 (3.1)	0.28 (0.09, 0.48)
	T2	10.1 (3.2)	10.8 (3.3)	0.26 (0.06, 0.45)
UWES-9 Dedication subscale score	T0	11.6 (3.2)	11.6 (3.5)	
[Possible score range: 0–18]	T1	11.5 (3.0)	12.0 (3.5)	0.23 (0.03, 0.42)
	T2	11.7 (3.2)	11.9 (3.4)	0.11 (−0.08, 0.30)
UWES-9 Absorption subscale score	T0	11.2 (3.1)	10.9 (3.2)	
[Possible score range: 0–18]	T1	11.2 (2.8)	11.7 (3.2)	0.24 (0.05, 0.44)
	T2	11.5 (2.9)	11.7 (3.0)	0.10 (−0.09, 0.30)
UWES-9 Total score	T0	32.7 (8.5)	32.4 (9.0)	
[Possible score range: 0–54]	T1	32.8 (7.8)	34.3 (8.8)	0.31 (0.11, 0.50)
	T2	33.3 (8.3)	34.5 (8.7)	0.19 (−0.01, 0.38)
Intention to leave				
ATS-12 Total score	T0	42.8 (11.1)	42.8 (12.2)	
[Possible score range: 12–84]	T1	42.1 (12.2)	41.0 (12.4)	0.15 (−0.05, 0.34)
	T2	44.2 (12.0)	41.7 (10.8)	0.25 (0.05, 0.44)
Employability				
SPE External employability subscale score	T0	22.5 (3.9)	21.8 (3.6)	
[Possible score range: 6–30]	T1	23.0 (3.6)	22.9 (3.5)	0.18 (−0.02, 0.37)
	T2	23.4 (3.5)	23.1 (3.7)	0.11 (−0.09, 0.30)
SPE Internal employability subscale score	T0	13.9 (2.5)	13.4 (2.8)	
[Possible score range: 4–20]	T1	14.2 (2.4)	14.1 (2.6)	0.17 (−0.02, 0.36)
	T2	14.4 (2.2)	14.1 (2.8)	0.01 (−0.19, 0.20)
SPE Total score	T0	40.1 (5.6)	38.8 (5.9)	
[Possible score range: 11–55]	T1	41.2 (5.2)	40.8 (5.6)	0.23 (0.03, 0.42)
	T2	41.8 (5.2)	41.0 (6.1)	0.11 (−0.09, 0.30)
Work performance				
IWPQ Task performance subscale score	T0	2.33 (0.82)	2.38 (0.85)	
[Possible score range: 0–4]	T1	2.45 (0.79)	2.45 (0.87)	−0.07 (−0.27, 0.12)
	T2	2.52 (0.83)	2.49 (0.86)	−0.14 (−0.33, 0.06)
IWPQ Contextual performance subscale score	T0	2.04 (0.85)	2.00 (0.93)	
[Possible score range: 0–4]	T1	2.13 (0.84)	2.15 (0.90)	0.12 (−0.07, 0.31)
	T2	2.12 (0.89)	2.16 (0.96)	0.16 (−0.04, 0.35)
IWPQ Counterproductive work behaviour subscale score	T0	1.45 (0.67)	1.50 (0.72)	
[Possible score range: 0–4]	T1	1.40 (0.70)	1.40 (0.64)	0.10 (−0.09, 0.30)
	T2	1.50 (0.69)	1.35 (0.61)	0.30 (0.10, 0.49)

### Effects of intervention on resilience

**Fig. 2:**
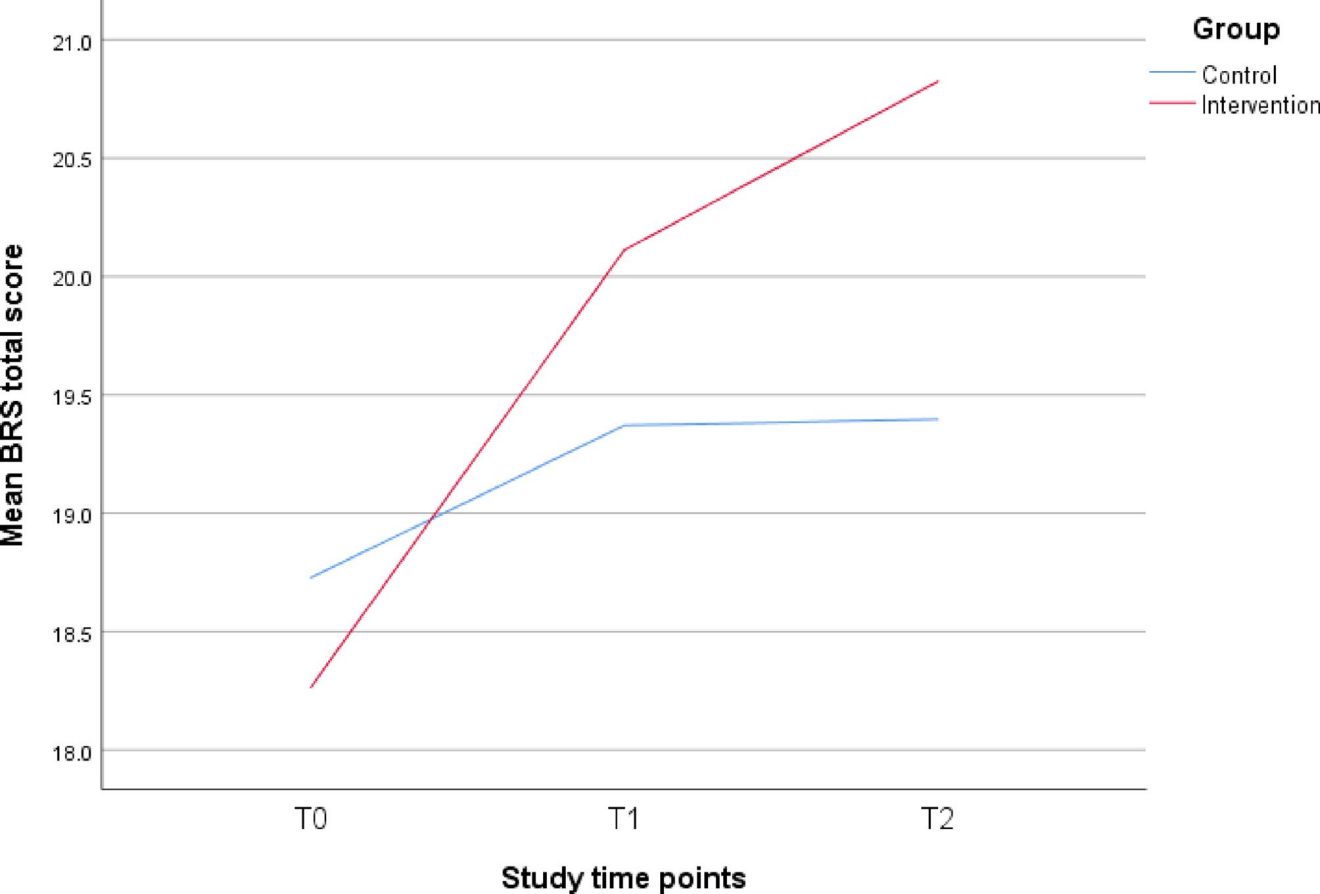
Line charts for brief resilience score (BRS) total score across the study time points.

The GEE analyses showed that participants in the intervention group, on average, had greater increments in the BRS total score at both T1 (regression coefficient of Group*T1, B = 1.22, 95% CI: 0.54 to 1.90, *P* < 0.001) and T2 (Group*T2, B = 1.77, 95% CI: 1.04 to 2.50, *P* < 0.001) with respect to T0 when compared with those in the control group (Table 3; Fig. 2). The Hedges’ *g* effect sizes were small to moderate at T1 (*g* = 0.38, 95% CI: 0.18 to 0.57) and T2 (*g* = 0.51, 95% CI: 0.32 to 0.71).

**Table 3:** Generalised estimating equations (GEE) models for the comparison of the outcomes across study time points between the control and intervention group. (Only the model estimates of regression coefficients of the dummy variables for the group [Group: 0 = Control (reference); 1 = Intervention]; T0 = at baseline; T1 = immediately after training; T2 = 3 months after training; Group*T1 and Group*T2 = time points and group interaction terms;. BRS = Brief resilience scale; UWES-9 = The 9-item Utrecht working engagement scale short version; ATS-12 = The 12-item anticipated turnover scale; SPE = Self-perceived employability scale; IWPQ = Individual work performance Questionnaire; B = unstandardized coefficient; *P < 0.05; **P < 0.01; ***P < 0.001).

Outcomes	Regression coefficients of the GEE models				
	Group	T1	T2	Group*T1	Group*T2
	B (95% CI)	B (95% CI)	B (95% CI)	B (95% CI)	B (95% CI)
Resilience					
BRS Total score	−0.46 (−1.26, 0.33)	0.60 (0.21, 0.99)**	0.71 (0.31, 1.12)**	1.22 (0.54, 1.90)***	1.77 (1.04, 2.50)***
Work engagement					
UWES-9 Vigour subscale score	0.01 (−0.64, 0.66)	0.06 (−0.28, 0.39)	0.20 (−0.17, 0.57)	0.65 (0.16, 1.13)***	0.68 (0.14, 1.22)*
UWES-9 Dedication subscale score	0.02 (−0.62, 0.67)	−0.03 (−0.34, 0.29)	0.17 (−0.18, 0.52)	0.52 (0.02, 1.02)*	0.25 (−0.29, 0.79)
UWES-9 Absorption subscale score	−0.29 (−0.89, 0.32)	0.12 (−0.21, 0.45)	0.44 (0.07, 0.81)*	0.68 (0.13, 1.24)*	0.37 (−0.20, 0.95)
UWES-9 Total score	−0.25 (−1.95, 1.45)	0.16 (−0.62, 0.95)	0.83 (−0.07, 1.72)	1.85 (0.57, 3.12)**	1.29 (−0.09, 2.66)
Intention to leave					
ATS-12 Total score	0.00 (−2.26, 2.26)	−0.56 (−1.68, 0.56)	1.42 (0.07, 2.77)*	−1.26 (−3.20, 0.67)	−2.60 (−4.77, −0.43)*
Employability					
SPE External employability subscale score	−0.67 (−1.40, 0.05)	0.56 (0.16, 0.96)**	0.93 (0.50, 1.36)***	0.52 (−0.08, 1.13)	0.34 (−0.36, 1.03)
SPE Internal employability subscale score	−0.44 (−0.95, 0.07)	0.34 (0.05, 0.62)*	0.62 (0.29, 0.95)***	0.34 (−0.09, 0.77)	0.05 (−0.46, 0.57)
SPE Total score	−1.33 (−2.44, −0.22)*	1.00 (0.41, 1.58)**	1.66 (0.99, 2.33)***	0.99 (0.09, 1.89)*	0.55 (−0.52, 1.63)
Work performance					
IWPQ Task performance subscale score	0.06 (−0.11, 0.22)	0.13 (0.04, 0.21)**	0.22 (0.12, 0.33)***	−0.05 (−0.19, 0.09)	−0.10 (−0.25, 0.05)
IWPQ Contextual performance subscale score	−0.03 (−0.21,0.14)	0.10 (0.01, 0.20)*	0.10 (−0.01, 0.20)	0.07 (−0.06, 0.20)	0.10 (−0.05, 0.24)
IWPQ Counterproductive work behavior subscale score	0.06 (−0.08, 0.19)	−0.05 (−0.13, 0.03)	0.05 (−0.04, 0.15)	−0.06 (−0.18, 0.07)	−0.20 (−0.34, −0.07)**

### Effects of intervention on work engagement

The GEE analyses revealed that the BRAW training could be effective in improving work engagement right after intervention (Table 3, Fig. S1). The Group*T1 interaction terms were all significant at T1 for all the subscales and total scores of the UWES (Group*T1, B ranged from 0.52 to 1.85, all *P* < 0.05), but only the effect on the vigor subscale could be sustainable at 3 months (Group*T2, B = 0.68, 95% CI: 0.14 to 1.22, *P* < 0.05).

### Effects of intervention on intention to leave, employability and work performance

Regarding the effects on the outcomes of intention to leave (Fig. S2), employability (Fig. S3), and work performance (Fig. S4), the intervention group demonstrated a significantly greater reduction in the ATS score at T2 (Group*T2, B = −2.60, 95% CI: −4.77 to −0.43, *P* < 0.05). A significantly greater increment in the SEP total score at T1 (Group*T1, B = 0.99, 95% CI: 0.09 to 1.89, *P* < 0.05) was found. A significantly greater reduction in the IWPQ counterproductive work behavior subscale score at T2 (Group*T2, B=−0.20, 95% CI: −0.34 to −0.07, *P* < 0.01) than in the control group. Table 3 presents the detailed GEE analysis results.

### Sensitivity analysis

Given the statistical differences in participants’ age and professional qualifications between groups, we conducted a sensitivity analysis by adjusting these characteristics in the GEE analyses for the outcomes. The adjusted GEE results (Table S5) were generally consistent with the results of crude GEE analyses, which support the robustness of the study results.

### Satisfaction with training

Most participants (163/193, 84.5%) completed all sessions of the BRAW. This result indicates a high level of engagement among participants. As shown in Table S6, the overall participants felt that the training content was good (62%) or excellent (17.4%). Most of them (72.0%) claimed that training matched their anticipation and needs (65%). Most of them (90.7%) will recommend it to friends. More than half were satisfied with the content (61.5%), and most (87.5%) thought the training could help them solve problems. The result showed that overall satisfaction with training was high (72.0%), and the majority of participants (78.9%) would like to revisit training.

## Discussion

This study developed and validated BRAW training for healthcare professionals. We used a 2-arm RCT to examine the effect of BRAW on resilience, work engagement, intention to leave, employability, and work performance. We found that the training had a positive impact on resilience, work engagement, intention to leave, and counterproductive work behavior at the post-intervention and/or 3-month follow-up.

Our study found that healthcare professionals experienced significant improvement in resilience following participation in the BRAW, as well as a 3-month follow-up assessment. This finding is consistent with previous studies^30,31^, such digital resilience trainings improved resilience level among American and Italian healthcare professionals. Because resilience is a modifiable factor, health professionals can acquire the necessary skills through BRAW videos and quizzes, reinforce them with homework, and use them to practice overcoming obstacles in clinical settings in Singapore. Resilient healthcare professionals may overcome hardship, conflict, and failure by being adaptable and resourceful^57^. They can adequately stand up for themselves, handle unforeseen events, and bounce back from adversity. Thus, BRAW can bolster resilience among healthcare professionals at post-intervention and 3-month follow-up assessments. Healthcare professionals can cope with the demands placed on them, especially when dealing with constantly changing priorities and a heavy workload in healthcare settings.

We discovered that BRAW significantly improved the total scale and all subscales of work engagement among healthcare professionals at post-intervention and, as well as the rigor subscale at the 3-month follow-up assessment, compared with healthcare professionals in the control group. A previous RCT on work engagement in Vietnam^32^ echoed these results. One possible explanation is that both trials used CBT concepts in their interventions. The acquisition of cognitive restructuring abilities enables participants to effectively address negative emotions and cultivate positive thinking on job-related obstacles, hence elevating their level of engagement at work^58^. The BRAW may be associated with improved personal and psychosocial job resources, potentially enhancing work engagement^57^. These resources, including self-efficacy and workplace social support, assist participants in reaching professional objectives and fostering personal growth^59^. After the intervention, the participants may have had a positive self-evaluation, and they may have felt confident in their ability to control and impact their environments successfully. They might be more engaged at work.

The vigor subscale continuously produced noteworthy findings during the 3-month follow-up, which may have something to do with the participants’ gradual development of emotion-regulation cognitive processes^60^. The fact that they perceived things favorably, concentrated on goals and acceptance, were prepared to put effort into their job, and persisted in the face of challenges implies that they had high levels of energy and mental resilience while working^32^.

We observed that participants in BRAW can decrease their intention to leave at 3-month follow-up when compared with the waitlist group, and this result was inconsistent with a previous study in the United States of America^33^. This discrepancy in these results might be attributed to differing sample sizes and the theoretical basis of the intervention. The control and intervention groups in our study had 217 and 193 participants, respectively, while in the prior study^33^, the corresponding numbers were 11 and 11. It is possible that a finding from a small sample size might not be strong enough to identify differences between the groups^61^. Another reason could be that BRAW used the concepts of CBT^18^, ACT^19^, and PST^20^, while the previous trial^33^ used a social constructivist philosophy. However, a previous study^62^ found a significant negative relationship between resilience and intervention. This result aligns with our current study, which found that resilient healthcare professionals are less likely to leave their organization. Therefore, the BRAW training could retain healthcare professionals.

The current study did not find significant changes in employability over time or differences between intervention and waitlist groups. This can be attributed to a global shortage of healthcare professionals, which resulted in high employability within healthcare settings^63^. Consequently, there may be less distinction between groups. However, employability is shaped by external factors, such as job market trends, organizational support, and leadership qualities^64^. Without modifications in these areas, individual intervention may have little impact. Hence, the employability of both groups was similar.

Additionally, compared to the control group, BRAW participants significantly reduced counterproductive work behaviors during the 3-month follow-up. Given that counterproductive work behavior can lead to poor patient care outcomes, low staff morale, and a reduction in the performance of organization^65^, BRAW demonstrated a significant effect on the elimination of counterproductive work behaviors in this study. One possible explanation might be that BRAW provided healthcare professionals with coping strategies, problem-solving abilities, and stress-reduction methods to prevent the potential negative effects of work stress and enhance performance in the workplace^66^. Learning from setbacks requires a positive attitude and emotion regulation abilities. Consequently, counterproductive work behaviors could be eliminated. However, we only observed significant reductions in counterproductive work behaviors, which were not present in other task performance, contextual performance, or adaptive performance subscales. Further investigation is necessary to understand the potential relationship between the distinct characteristics of each performance.

This study found some strengths. The BRAW is a theory-driven intervention that draws from the theoretical foundations of CBT^18^, ACT^19^, and PST^20^. The design was evidence-based because our previous systematic review^27^ provided evidence to guide the features of training for healthcare professionals. A total of 378 healthcare professionals were involved in this RCT, and a relatively large sample size could provide more reliable results because they have smaller margins of error and lower standards of deviation^67^. The sample, which was recruited from a variety of social media, suggests that the BRAW could be useful for a diverse healthcare workforce. The high completion rate and satisfactory feedback from the participants indicated that the BRAW has the advantages of greater accessibility, user-friendliness, and ease of use.

While the current study’s findings are intriguing, it is important to consider several methodological limitations when interpreting the results. First, we used a convenience sample, and our healthcare professionals were a female-dominated group, limiting the generalizability of the findings to gender-balanced occupational groups in healthcare settings. Second, we only included self-reported results that might lead to social desirability bias, which is another limitation. Third, the diversity of cultures, the nature of work, and individual differences made it difficult to apply a single resilience training program to all healthcare organizations across different countries. While Singapore encompasses both Eastern and Western cultures, we anticipate tailoring the intervention to the specific context of each country. Fourth, this study measured the outcomes at post-intervention and 3-month follow-up assessments. We did not gather long follow-up information, and the sustainability of the intervention is uncertain. Fifth, we used a waitlist as a comparator; the participants in the control group could have high expectations for the intervention, and it might both inflate the intervention and deflate the control^68^. Finally, an imbalance in the number of participants between groups at baseline can reduce the power of statistical tests, making it harder to identify a true effect of the intervention^69^.

We developed BRAW to enhance resilience, work engagement, intention to leave, and counterproductive work behaviors among healthcare professionals, including physicians, nurses, allied health professionals, and others. The BRAW was a 6-session web-based and self-paced resilience training program that could provide cost-effective support for healthcare professionals, particularly when they encountered the complexity of healthcare settings. Resilient healthcare professionals should understand their strengths and weaknesses, seize opportunities, and navigate uncertainty and changes^70^. Nonetheless, cooperation between stakeholders and policymakers is necessary for successful dissemination and implementation. Therefore, BRAW can be viewed as supplementary training that helps healthcare professionals prepare for managing unpredictable pandemics in the future. Considering the BRAW training is an effective, scalable, and practical means of delivering online resilience training, this training can be applied to other employees in different workplaces to build their resilience to handle unexpected situations and overcome hardship.

Further trials could explore the use of objective measures of resilience outcomes, utilizing toolkits based on external observations^71^. Future research should adopt a placebo or active comparator to provide greater scientific reliability for results^72^. Future research trials can incorporate extended follow-ups beyond 3 months, which would be beneficial to examine the long-term impacts of the BRAW. Further studies might replicate the current study in different countries to compare the results across different cultures and increase confidence in the validity and generalization of findings.

## Conclusion

In summary, the results of this study contribute to the literature on online resilience training for healthcare professionals by providing evidence to enhance resilience, improve work engagement, decrease intention to leave and eliminate counterproductive work behavior. To improve the robustness of the study, further research should improve its methodological design, which includes objective measures, active comparators, and long-term follow-up. Healthcare professionals should be better equipped to handle unpredictable pandemics in the future.

## Supplementary Information

Below is the link to the electronic supplementary material.

Supplementary Material 1

## Data Availability

The data that support the findings of this study are available from the corresponding author upon request.
