# Supplementary material for: A randomized controlled trial investigating digital resilience training for healthcare professionals
Source: Sci Rep. 2025 Dec 24;15:44514. doi: 10.1038/s41598-025-28028-z (PMC12739135; doi:10.1038/s41598-025-28028-z)
Supplement: Supplementary file 1 — Supplementary Material 1 [file 41598_2025_28028_MOESM1_ESM.docx]

| **Sessions** | **Objectives** | **Content** | **Components** |
| --- | --- | --- | --- |
| 1. Happiness and positivity | - To understand the significance of happiness and positivity in healthcare settings - To describe the concepts of happiness and positivity - To build happiness and positivity in the workplace | - Significance of happiness and positivity - Concepts of happiness and positivity - Building happiness and positivity in the workplace | - 2 short videos (6 to 8 minutes) - 3 short quizzes - Session 1 forum - 2 homework exercises (Ways and activities to boost happiness) |
| 1. Cognitive restructuring | - To identify dysfunctional automatic thoughts - To apply cognitive restructuring techniques - To use rational responses to automatic thoughts | - Introduction of dysfunctional automatic thoughts - Apply cognitive restricting techniques - Rational responses to automatic thoughts | - 2 short videos (6 to 8 minutes) - 3 short quizzes - Session 2 forum - 1 homework exercise (Thought record) |
| 1. Behavioral activation | - To identify meaningful activities - To deal with low motivation - To create a SMART action plan | - Potential activities - Motivation tips - Action plan | - 2 short videos (6 to 8 minutes) - 3 short quizzes - Session 3 forum - 1 homework exercise (SMART goal setting) |
| 1. Emotion regulation | - To prevent and manage conflict - To master the emotion regulation technique | - Courses of conflict - Conflict management - Emotion regulation techniques | - 2 short videos (6 to 8 minutes) - 3 short quizzes - Session 4 forum - 1 homework exercise (Identify and regulate a negative emotion) |
| 1. Positive work climate | - To build a great culture in a healthcare organization - To promote coworker relationship - To foster a positive work environment | - Reasons and ways for creating a great culture - Improve the relationship with coworkers - Individual efforts to foster a positive work environment | - 2 short videos (6 to 8 minutes) - 3 short quizzes - Session 1 forum - 1 homework exercise (creating a more positive work environment) |
| 1. Problem solving | - To identify the steps of problem-solving techniques - To use different approaches for solving problems in healthcare - To learn how to apply some ways for maintaining a work-life balance | - Problem-solving techniques - Different approaches to solve problems in healthcare - Maintaining work-life balance | - 2 short videos (6 to 8 minutes) - 3 short quizzes - Session 1 forum - 1 homework exercise (Using SOLVE framework for problem solving) |

**Supplementary Table S1.** Online session content of web-based building resilience at work (BRAW) training.

| **Week** | **Topic** | **Quiz, Forum, and Homework** |
| --- | --- | --- |
| 1 | **Happiness and positivity**  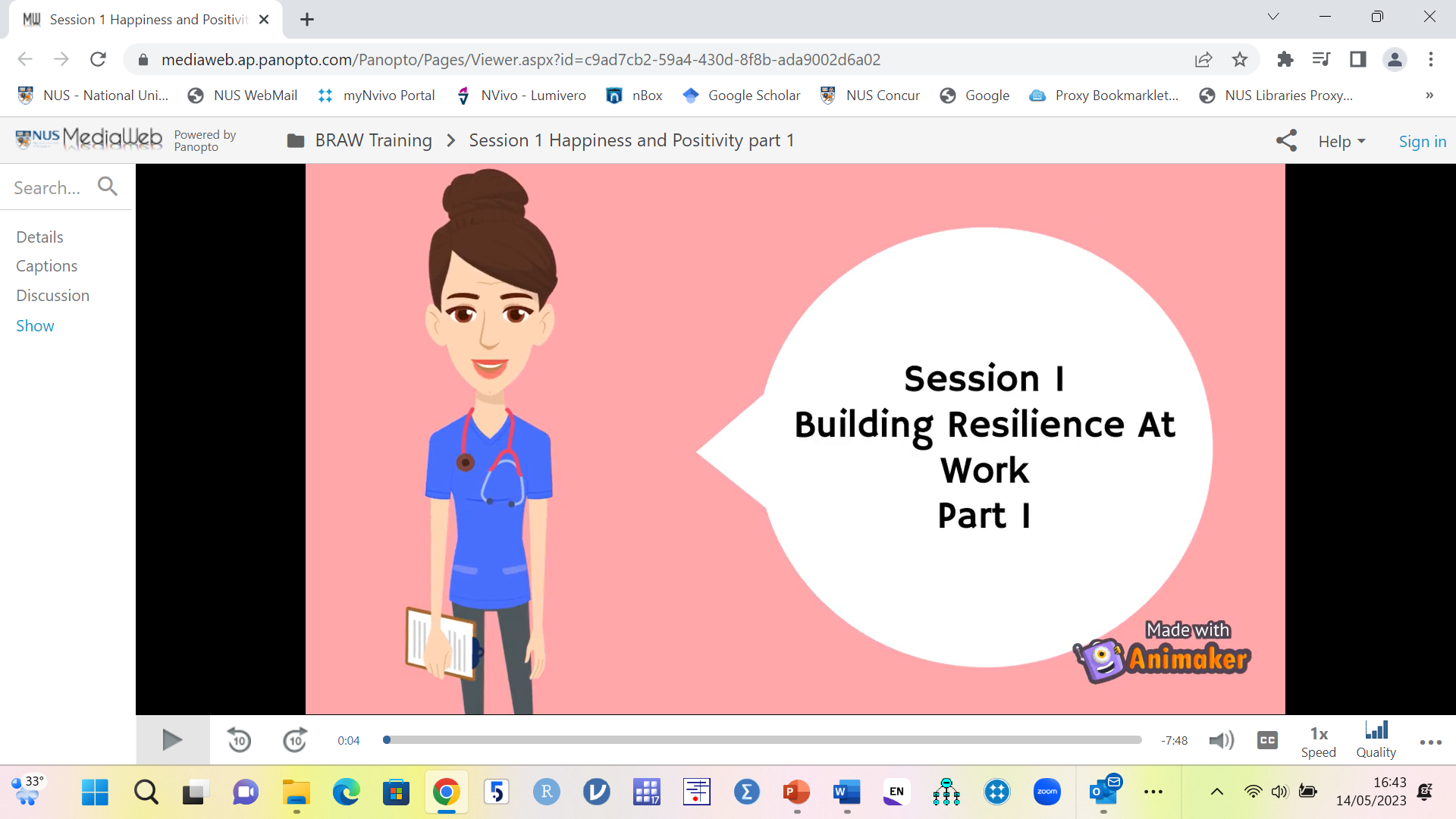 | **Quiz sample**  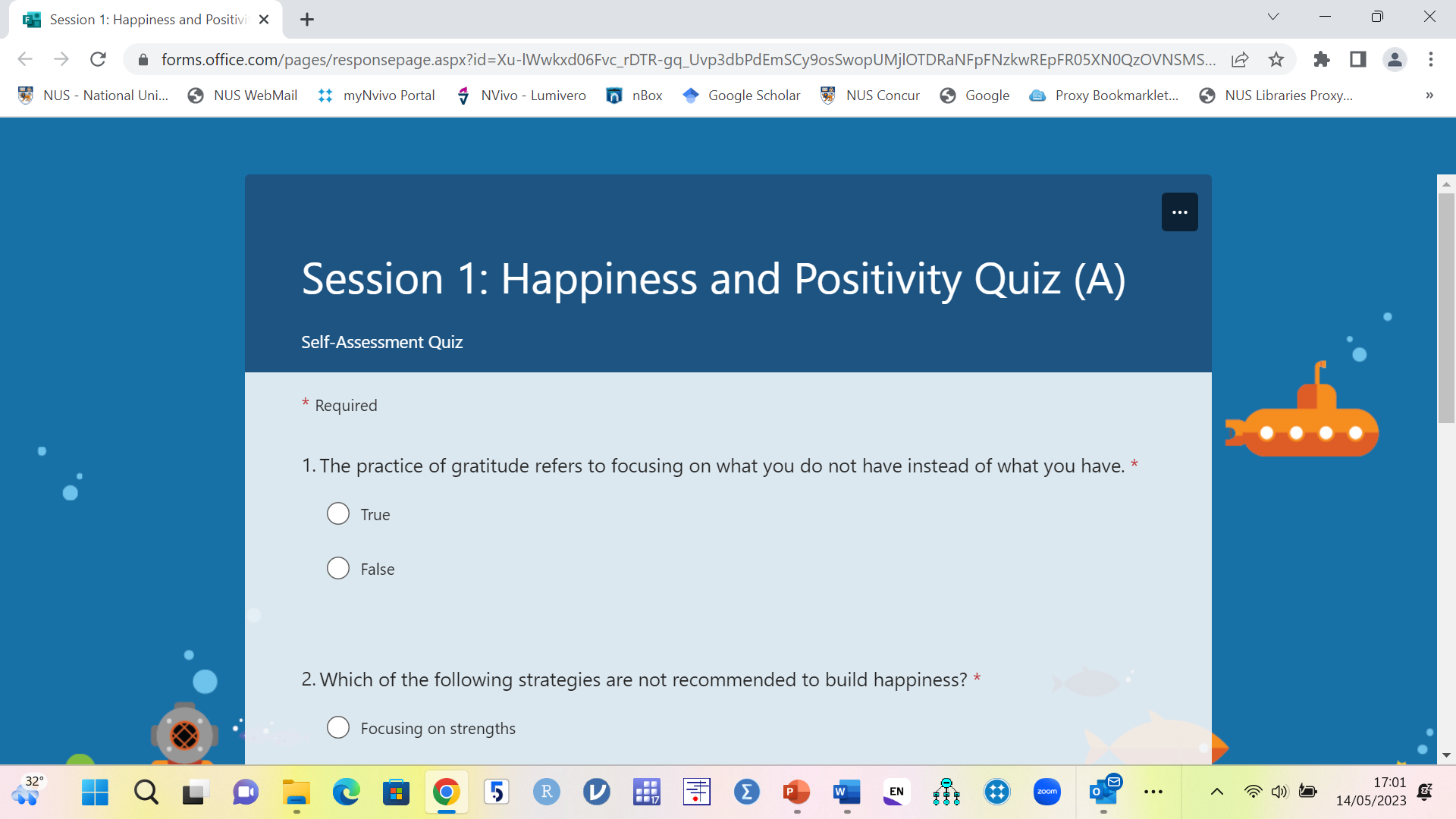 |
| 2 | **Cognitive restructuring**  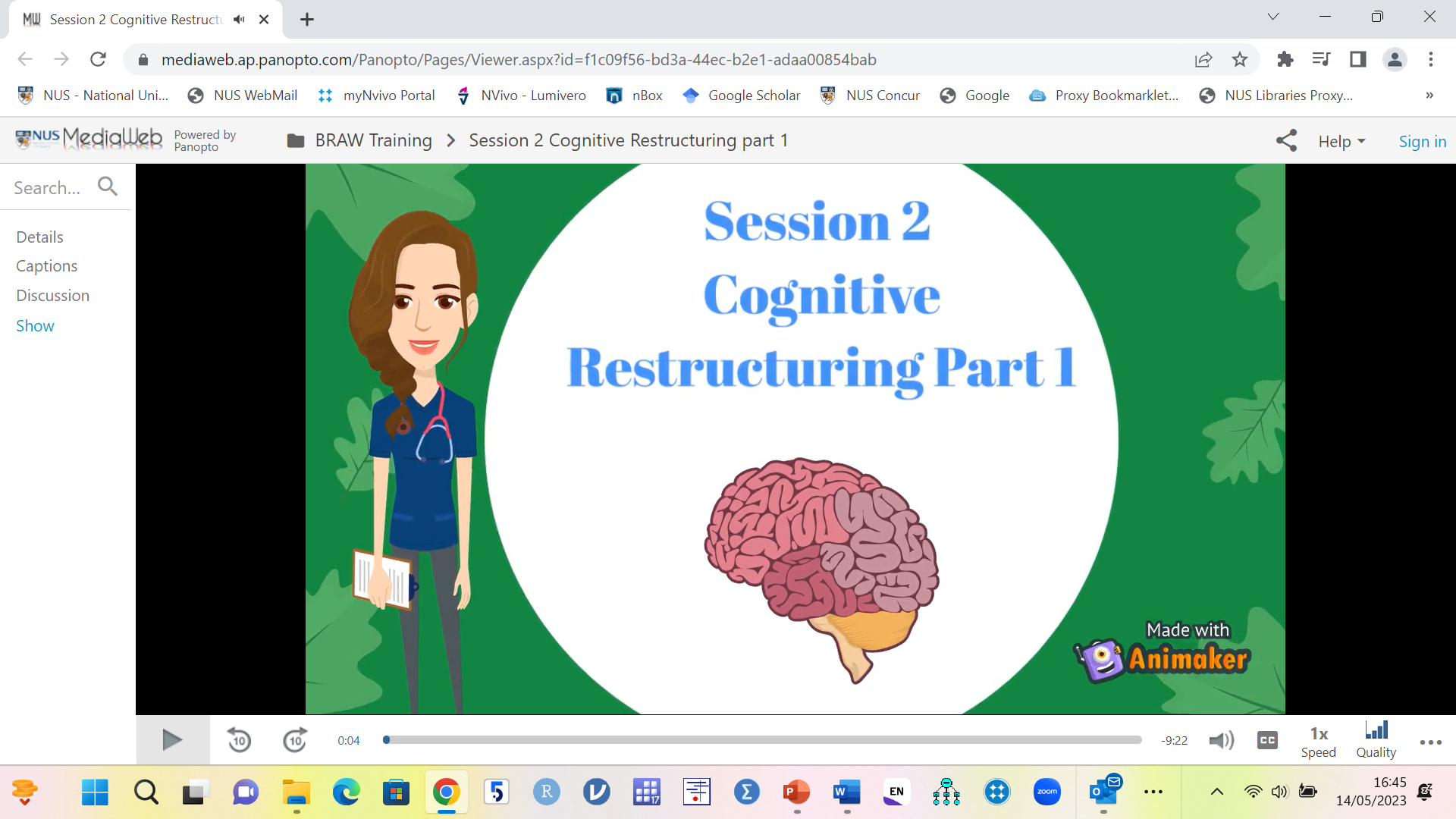 | **Forum sample**  **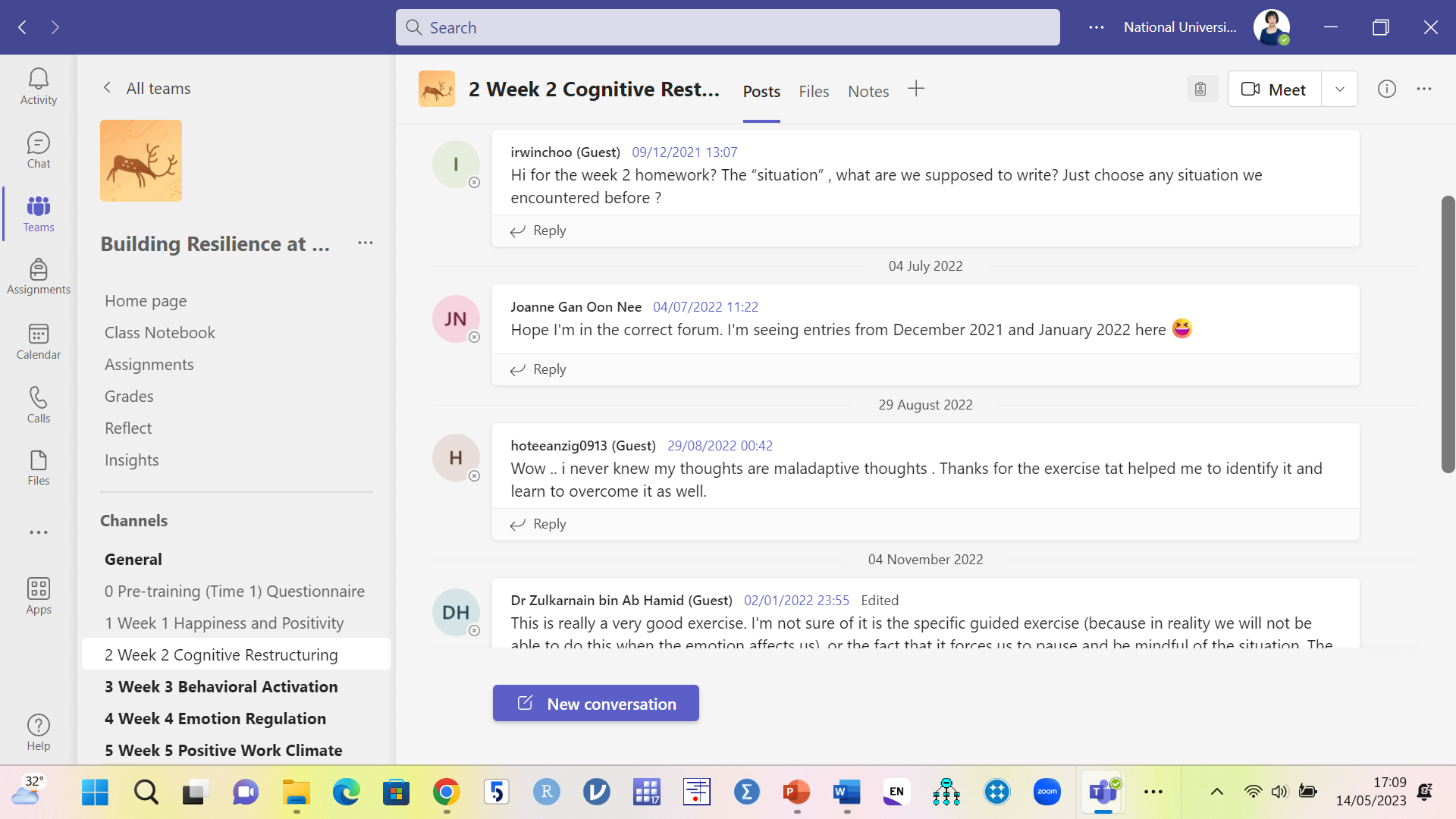** |
| 3 | **Behavioral activation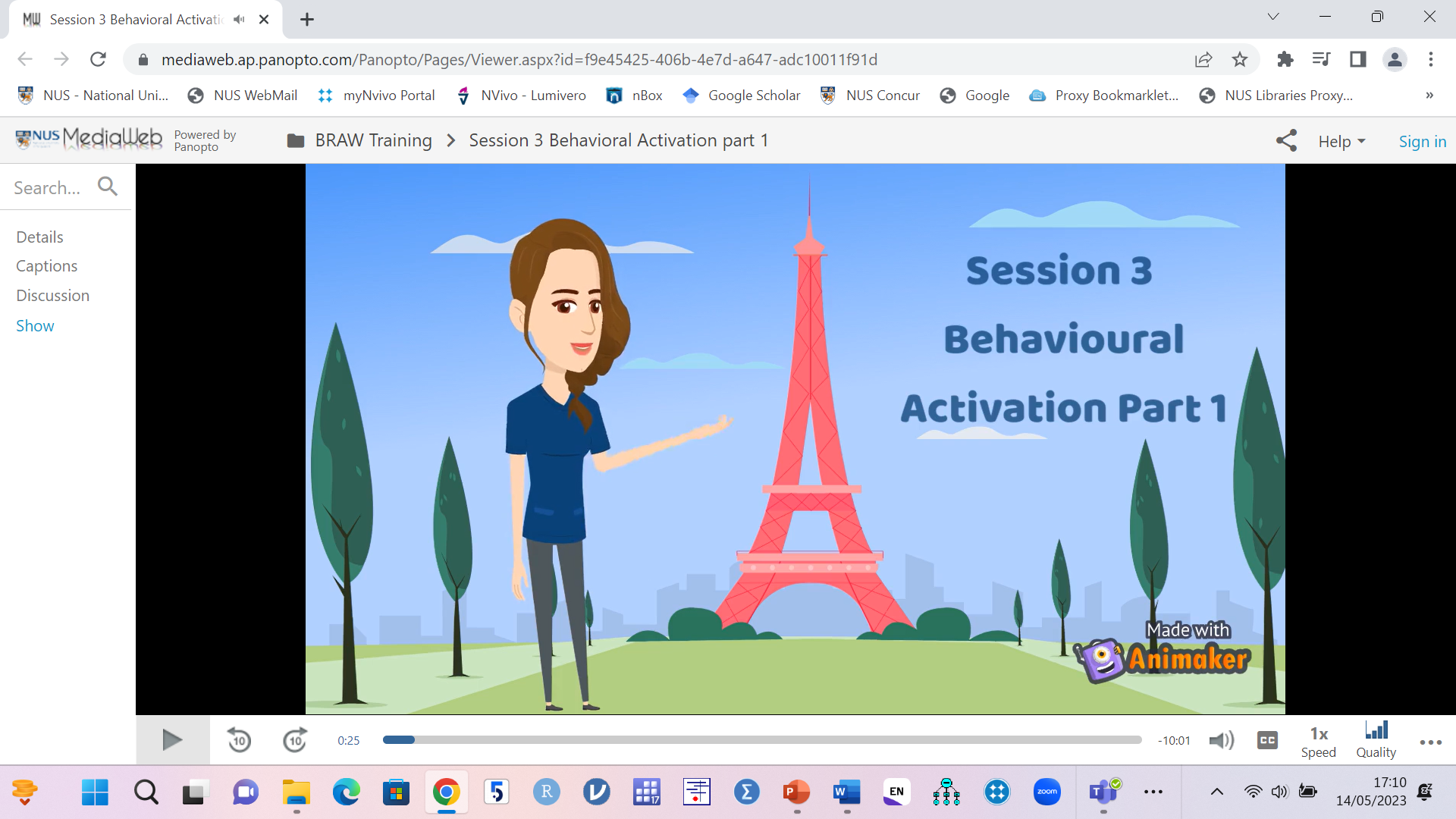** | **Homework sample**  **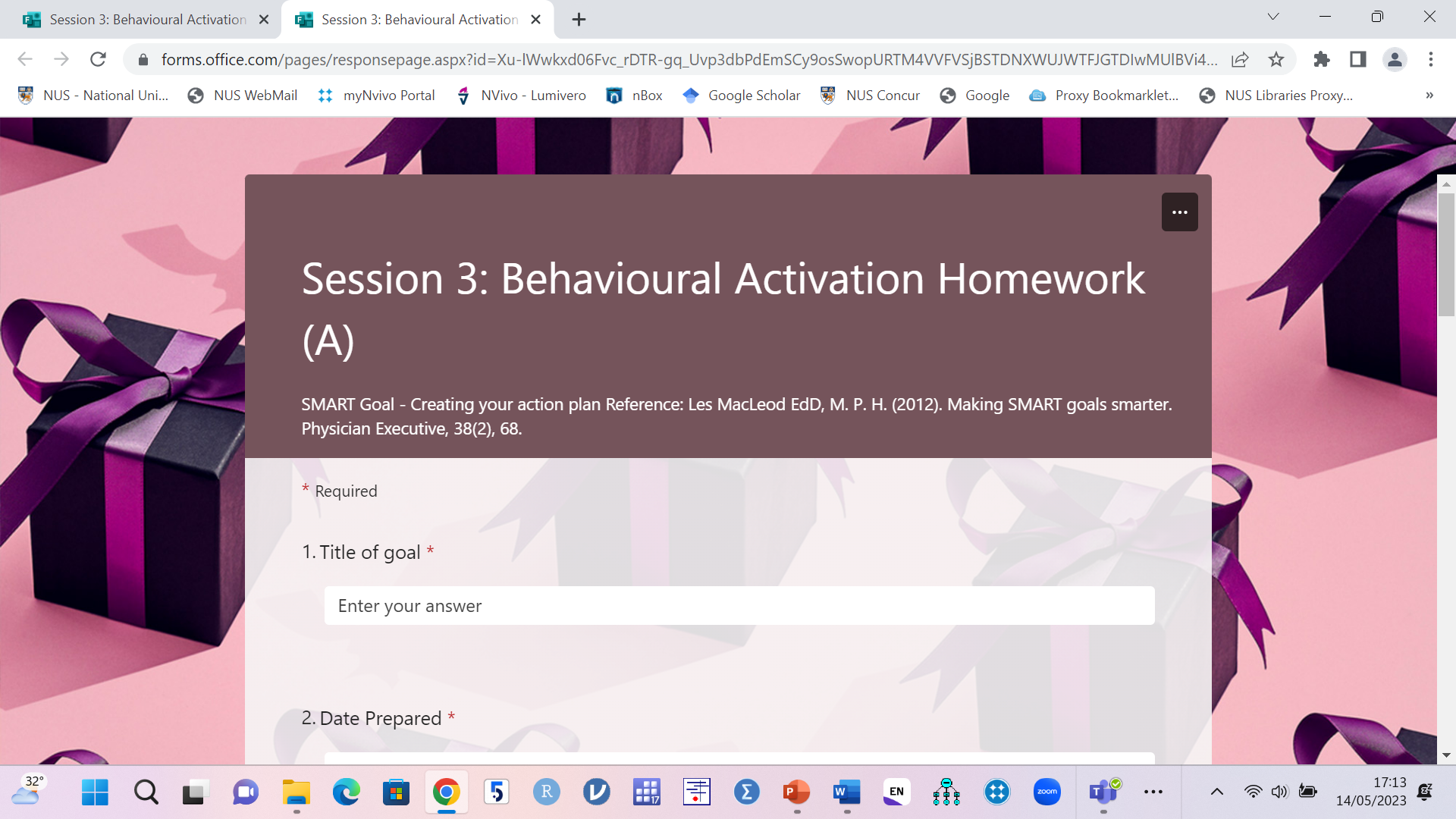** |

| 4 | **Emotion regulation**  **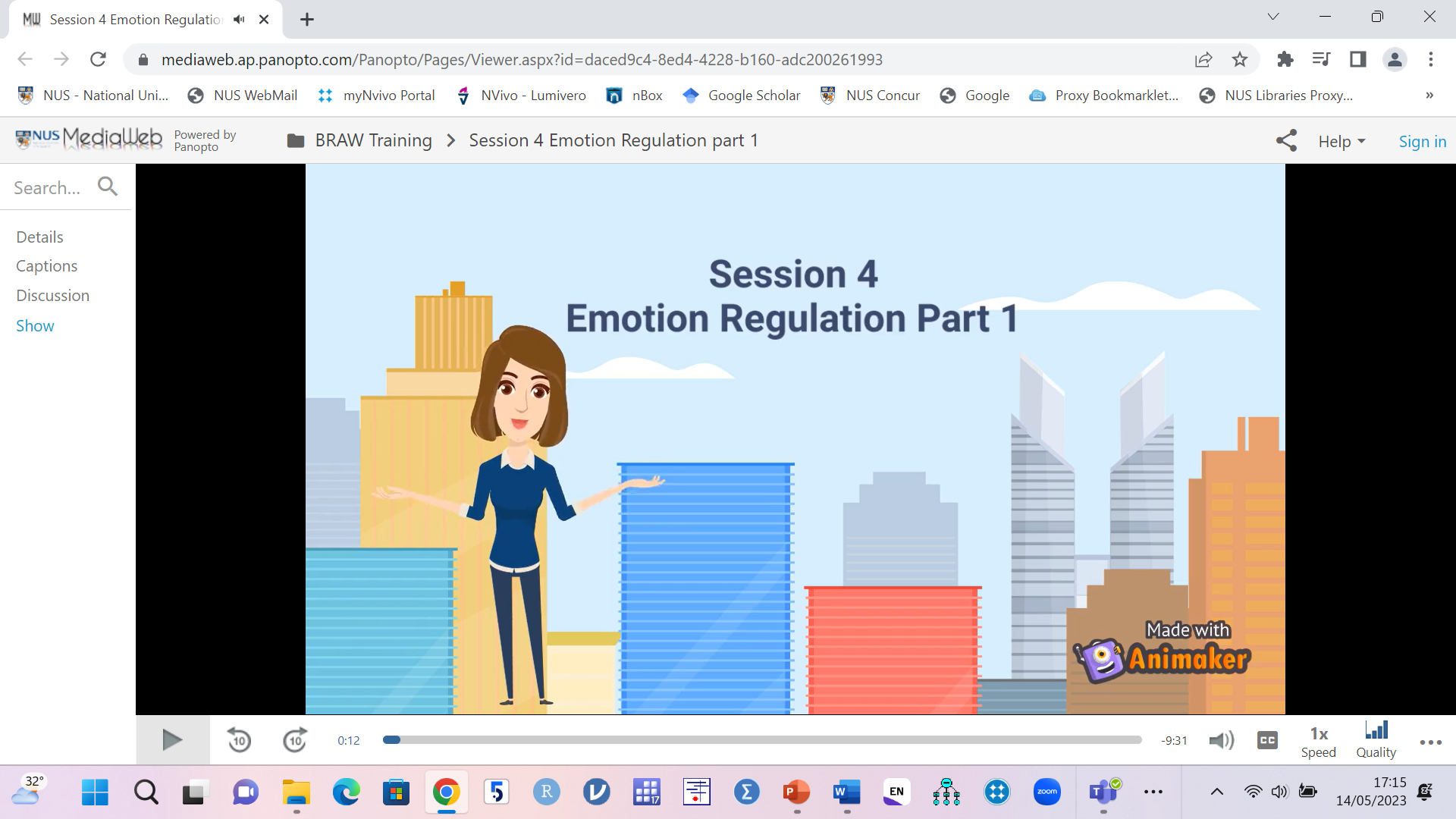** | **Quiz sample**  **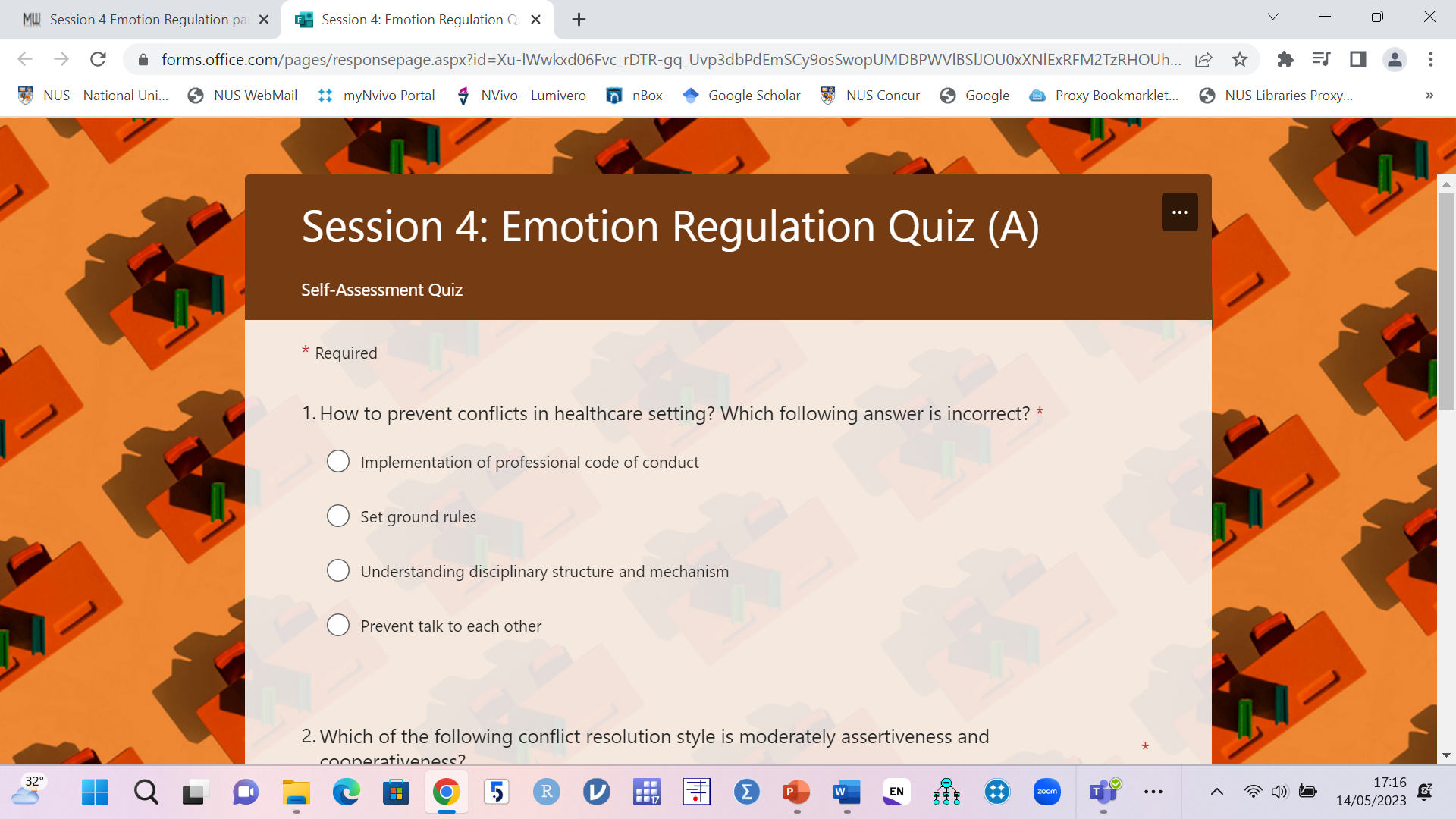** |
| --- | --- | --- |
| 5 | **Positive work climate**  **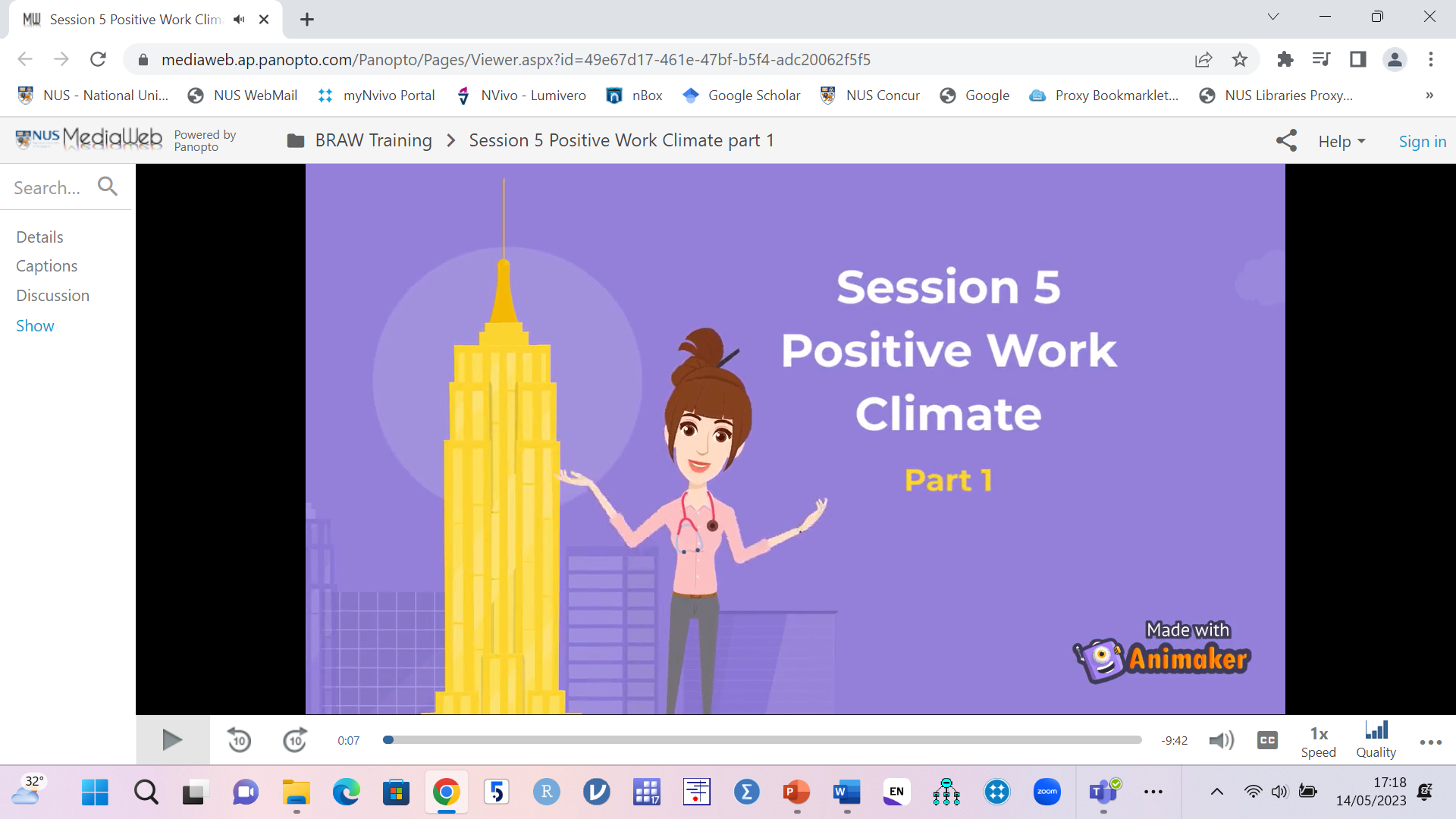** | **Forum sample**  **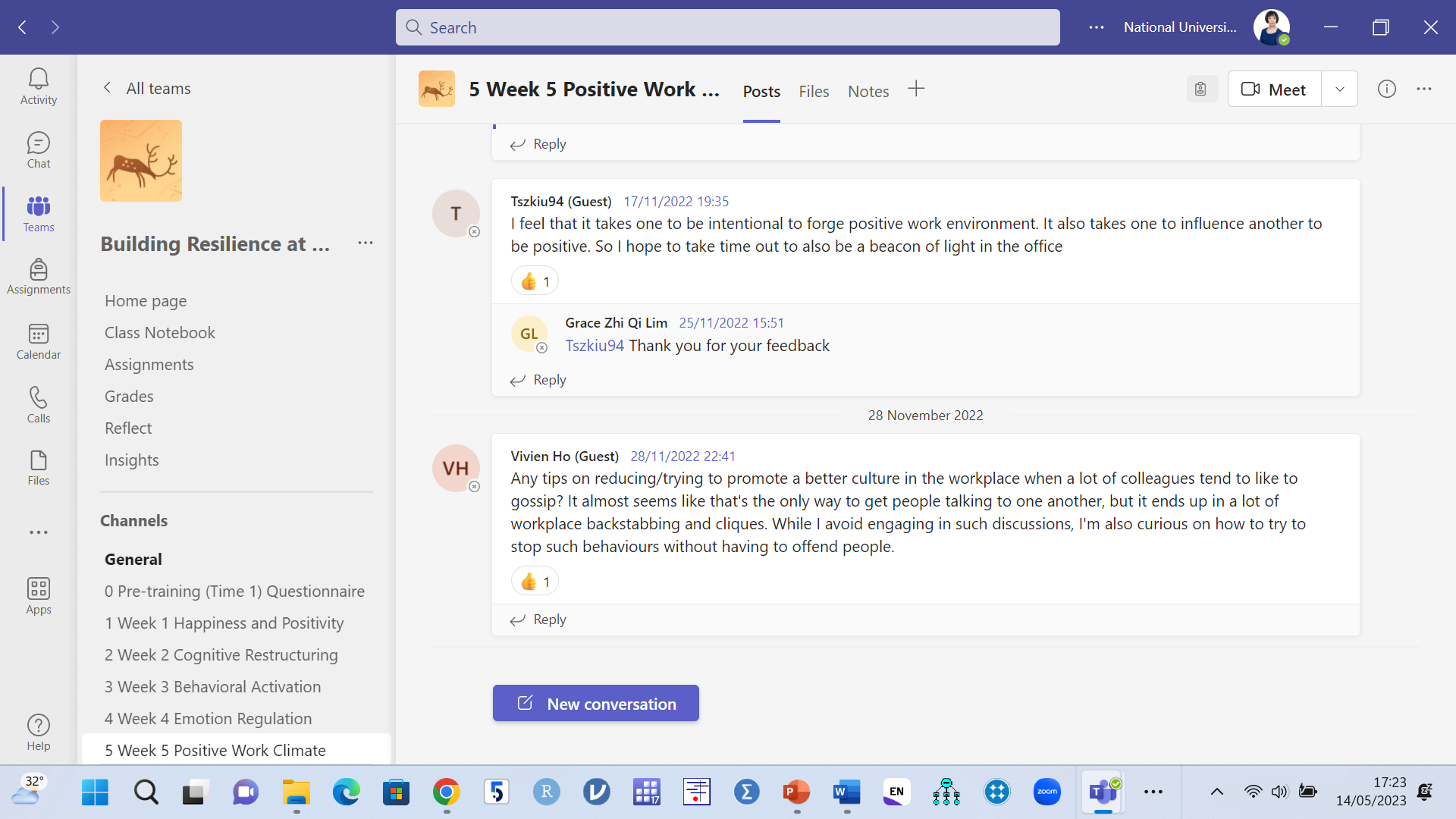** |
| 6 | **Problem-solving**  **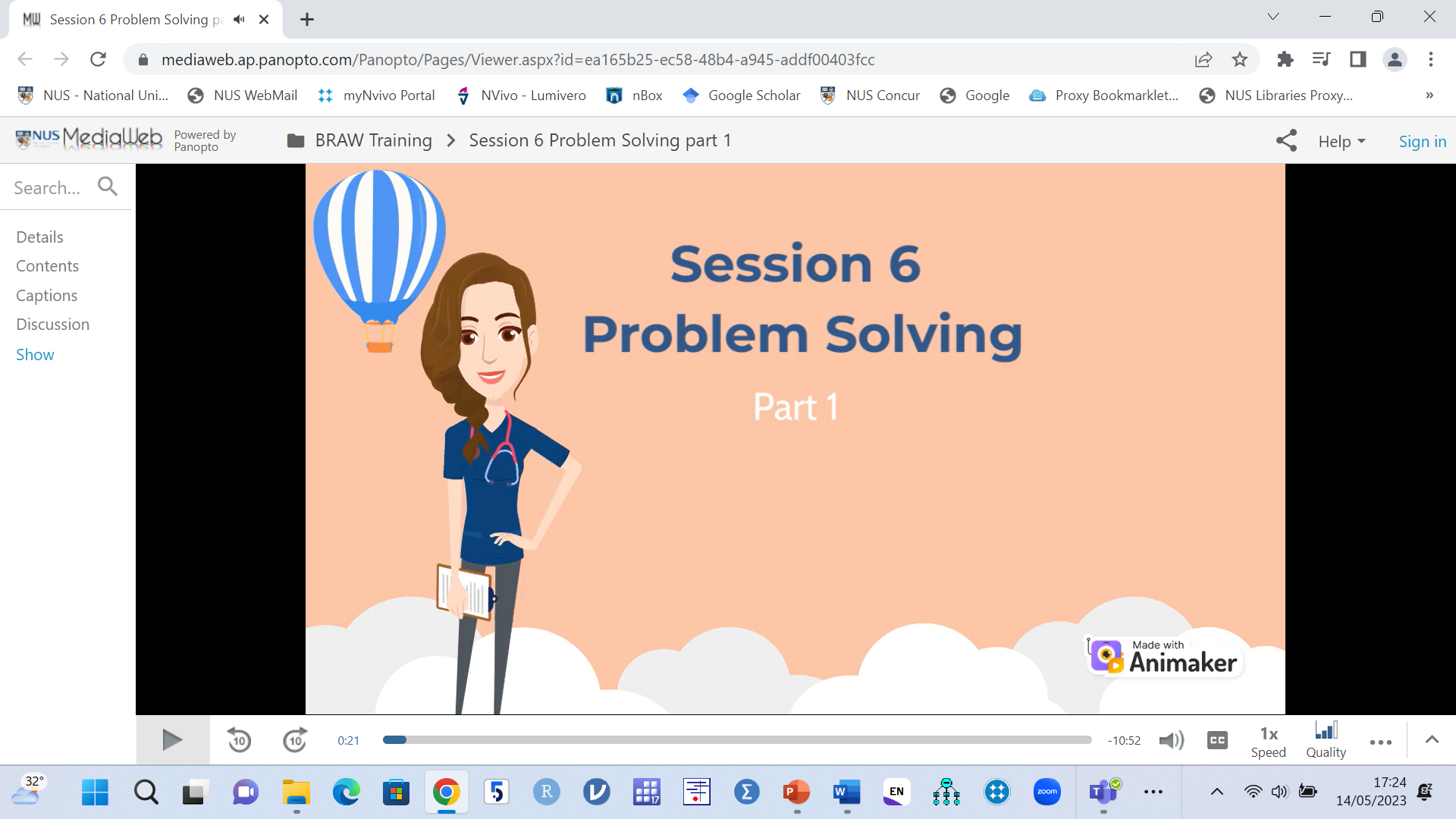** | **Homework sample**  **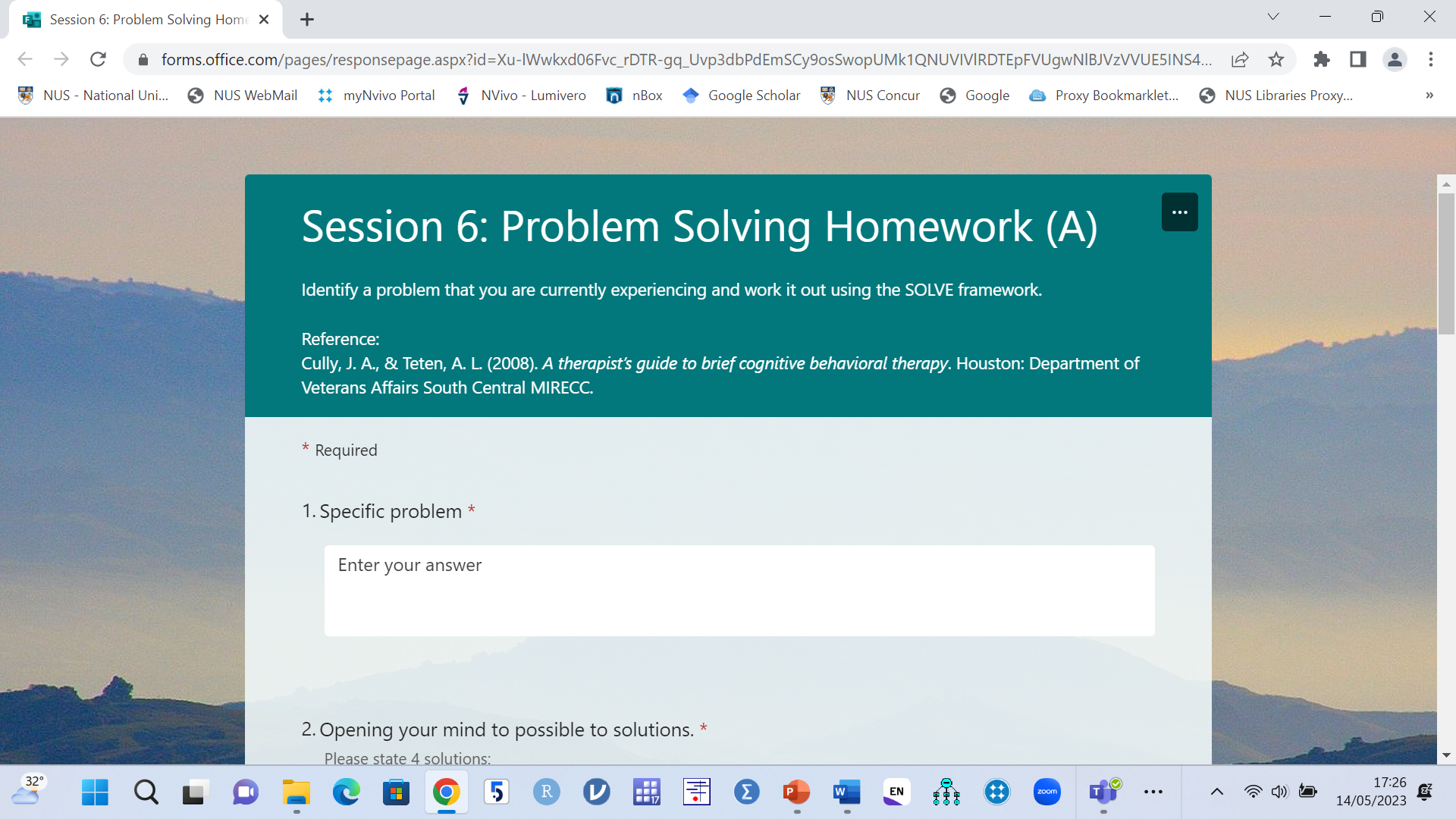** |

**Supplementary Table S2.** Screenshots of six weekly web-based building resilience at work (BRAW) training.

| **Section/**  **Topic** | **Item No** | **Checklist item** | **Reported on page No** |
| --- | --- | --- | --- |
| **Title and abstract** | | | |
|  | 1a | Identification as a randomised trial in the title | 1 |
|  | 1b | Structured summary of trial design, methods, results, and conclusions (for specific guidance see CONSORT for abstracts) | 3 |
| **Introduction** | | | |
| Background and objectives | 2a | Scientific background and explanation of rationale | 4-8 |
|  | 2b | Specific objectives or hypotheses | 8 |
| **Methods** | | | |
| Trial design | 3a | Description of trial design (such as parallel, factorial) including allocation ratio | 10, 11 |
|  | 3b | Important changes to methods after trial commencement (such as eligibility criteria), with reasons | N/A |
| Participants | 4a | Eligibility criteria for participants | 10 |
|  | 4b | Settings and locations where the data were collected | 11 |
| Interventions | 5 | The interventions for each group with sufficient details to allow replication, including how and when they were actually administered | 9, 12 |
| Outcomes | 6a | Completely defined pre-specified primary and secondary outcome measures, including how and when they were assessed | 12-14 |
|  | 6b | Any changes to trial outcomes after the trial commenced, with reasons | N/A |
| Sample size | 7a | How sample size was determined | 10 |
|  | 7b | When applicable, explanation of any interim analyses and stopping guidelines | N/A |
| Randomisation: |  |  |  |
| Sequence generation | 8a | Method used to generate the random allocation sequence | 11 |
|  | 8b | Type of randomisation; details of any restriction (such as blocking and block size) | 11 |
| Allocation concealment mechanism | 9 | Mechanism used to implement the random allocation sequence (such as sequentially numbered containers), describing any steps taken to conceal the sequence until interventions were assigned | 11 |
| Implementation | 10 | Who generated the random allocation sequence, who enrolled participants, and who assigned participants to interventions | 11 |
| Blinding | 11a | If done, who was blinded after assignment to interventions (for example, participants, care providers, those assessing outcomes) and how | 11 |
|  | 11b | If relevant, description of the similarity of interventions | N/A |
| Statistical methods | 12a | Statistical methods used to compare groups for primary and secondary outcomes | 15-16 |
|  | 12b | Methods for additional analyses, such as subgroup analyses and adjusted analyses | 15-16 |
| **Results** | | | |
| Participant flow (a diagram is strongly recommended) | 13a | For each group, the numbers of participants who were randomly assigned, received intended treatment, and were analysed for the primary outcome | 17-18 |
|  | 13b | For each group, losses and exclusions after randomisation, together with reasons | 17-18, Table S4 |
| Recruitment | 14a | Dates defining the periods of recruitment and follow-up | 17-18 |
|  | 14b | Why the trial ended or was stopped | N/A |
| Baseline data | 15 | A table showing baseline demographic and clinical characteristics for each group | 17, Table 1 |
| Numbers analysed | 16 | For each group, number of participants (denominator) included in each analysis and whether the analysis was by original assigned groups | 17 |
| Outcomes and estimation | 17a | For each primary and secondary outcome, results for each group, and the estimated effect size and its precision (such as 95% confidence interval) | 18-20 |
|  | 17b | For binary outcomes, presentation of both absolute and relative effect sizes is recommended | 18-20 |
| Ancillary analyses | 18 | Results of any other analyses performed, including subgroup analyses and adjusted analyses, distinguishing pre-specified from exploratory | 19-20 |
| Harms | 19 | All important harms or unintended effects in each group (for specific guidance see CONSORT for harms) | N/A |
| **Discussion** | | | |
| Limitations | 20 | Trial limitations, addressing sources of potential bias, imprecision, and, if relevant, multiplicity of analyses | 24 |
| Generalisability | 21 | Generalisability (external validity, applicability) of the trial findings | 24-25 |
| Interpretation | 22 | Interpretation consistent with results, balancing benefits and harms, and considering other relevant evidence | 20-23 |
| **Other information** | | |  |
| Registration | 23 | Registration number and name of trial registry | 1, 10 |
| Protocol | 24 | Where the full trial protocol can be accessed, if available | N/A |
| Funding | 25 | Sources of funding and other support (such as supply of drugs), role of funders | 1 |

**Supplementary Table S3.** CONSORT checklist of information to include when reporting a randomized trial.

|  | **Completers (N=343)** | **Dropouts (N=67)** | ***P*** |
| --- | --- | --- | --- |
| Age (years) |  |  |  |
| ≤ 25 | 81 (23.6%) | 15 (22.4%) | 0.86 |
| 26 – 30 | 91 (26.5%) | 19 (28.4%) |  |
| 31 – 40 | 121 (35.3%) | 21 (31.3%) |  |
| > 40 | 50 (14.6%) | 12 (17.9%) |  |
| Sex |  |  |  |
| Female | 57 (16.6%) | 14 (20.9%) | 0.40 |
| Male | 286 (83.4%) | 53 (79.1%) |  |
| Marital status |  |  |  |
| Married | 213 (62.1%) | 38 (56.7%) | 0.41 |
| Single/divorced/others | 130 (37.9%) | 29 (43.3%) |  |
| Ethnicity |  |  |  |
| Chinese | 285 (83.1%) | 54 (80.6%) | 0.86 |
| Malay | 26 (7.6%) | 5 (7.5%) |  |
| Indian | 25 (7.3%) | 7 (10.4%) |  |
| Others | 7 (2.0%) | 1 (1.5%) |  |
| Profession |  |  |  |
| Physician | 22 (6.4%) | 6 (9.0%) | 0.35 |
| Nurse | 161 (46.9%) | 37 (55.2%) |  |
| Allied health professional | 101 (29.4%) | 17 (25.4%) |  |
| Others | 59 (17.2%) | 7 (10.4%) |  |
| Professional qualification |  |  |  |
| Certificate/Diploma | 43 (12.5%) | 11 (16.4%) | 0.67 |
| Bachelor’s degree | 231 (67.3%) | 44 (65.7%) |  |
| Master’s degree or higher | 69 (20.1%) | 12 (17.9%) |  |
| Area of work |  |  |  |
| Hospital | 343 (100.0%) | 67 (100.0%) | 0.99 |
| Years of experience as healthcare professional |  |  |  |
| < 1 | 51 (14.9%) | 8 (11.9%) | 0.17 |
| 1 – 2 | 42 (12.2%) | 7 (10.4%) |  |
| > 2 – 5 | 75 (21.9%) | 17 (25.4%) |  |
| > 5 – 10 | 63 (18.4%) | 18 (26.9%) |  |
| > 10 – 20 | 87 (25.4%) | 9 (13.4%) |  |
| > 20 | 25 (7.3%) | 8 (11.9%) |  |
| Years in institution |  |  |  |
| < 1 | 70 (20.4%) | 10 (14.9%) | 0.43 |
| 1 – 2 | 52 (15.2%) | 17 (25.4%) |  |
| > 2 – 5 | 95 (27.7%) | 16 (23.9%) |  |
| > 5 – 10 | 64 (18.7%) | 11 (16.4%) |  |
| > 10 – 20 | 48 (14.0%) | 11 (16.4%) |  |
| > 20 | 14 (4.1%) | 2 (3.0%) |  |

**Supplementary Table S4.** Baseline characteristics between those who dropped out from the study and those who had not. [Variables marked with ^†^ are presented as mean (standard deviation), otherwise as frequency (%)].

|  | **Regression coefficients of the GEE models^#^** | | | | |
| --- | --- | --- | --- | --- | --- |
|  | **Group** | **T1** | **T2** | **Group*T1** | **Group*T2** |
| **Outcomes** | **B (95% CI)** | **B (95% CI)** | **B (95% CI)** | **B (95% CI)** | **B (95% CI)** |
| Resilience |  |  |  |  |  |
| BRS Total score | -0.40 (-1.19, 0.38) | 0.61 (0.22, 1.00)** | 0.72 (0.31, 1.13)** | 1.21 (0.53, 1.89)*** | 1.76 (1.03, 2.49)*** |
| Work engagement |  |  |  |  |  |
| UWES-9 Vigor subscale score | 0.04 (-0.59, 0.66) | 0.06 (-0.27, 0.40) | 0.20 (-0.17, 0.57) | 0.65 (0.16, 1.14)** | 0.69 (0.14, 1.23)* |
| UWES-9 Dedication subscale score | 0.06 (-0.57, 0.68) | -0.03 (-0.34, 0.29) | 0.17 (-0.18, 0.52) | 0.53 (0.03, 1.03)* | 0.26 (-0.29, 0.80) |
| UWES-9 Absorption subscale score | -0.24 (-0.85, 0.36) | 0.12 (-0.21, 0.45) | 0.44 (0.07, 0.81)* | 0.68 (0.12, 1.24)* | 0.37 (-0.20, 0.95) |
| UWES-9 Total score | -0.15 (-1.80, 1.50) | 0.17 (-0.62, 0.95) | 0.83 (-0.07, 1.72) | 1.85 (0.58, 3.13)** | 1.30 (-0.07, 2.67) |
| Intention to leave |  |  |  |  |  |
| ATS-12 Total score | -0.18 (-2.41, 2.06) | -0.57 (-1.69, 0.55) | 1.42 (0.07, 2.77)* | -1.26 (-3.20, 0.67) | -2.61 (-4.77, -0.45)* |
| Employability |  |  |  |  |  |
| SPE External employability subscale score | -0.64 (-1.37, 0.09) | 0.55 (0.15, 0.96)** | 0.92 (0.49, 1.35)*** | 0.53 (-0.07, 1.14) | 0.35 (-0.35, 1.05) |
| SPE Internal employability subscale score | -0.29 (-0.79, 0.21) | 0.34 (0.05, 0.62)* | 0.62 (0.28, 0.95)*** | 0.34 (-0.09, 0.77) | 0.05 (-0.46, 0.57) |
| SPE Total score | -1.14 (-2.26, -0.02)* | 0.99 (0.41, 1.58)** | 1.65 (0.98, 2.32)*** | 1.00 (0.09, 1.90)* | 0.57 (-0.51,1.65) |
| Work performance |  |  |  |  |  |
| IWPQ Task performance subscale score | 0.04 (-0.12, 0.20) | 0.13 (0.04, 0.21)** | 0.22 (0.12, 0.33)*** | -0.05 (-0.19, 0.09) | -0.10 (-0.25, 0.05) |
| IWPQ Contextual performance subscale score | 0.00 (-0.16, 0.17) | 0.11 (0.01, 0.20)* | 0.10 (-0.01, 0.20) | 0.07 (-0.06, 0.20) | 0.10 (-0.05, 0.24) |
| IWPQ Counterproductive work behavior subscale score | 0.06 (-0.08, 0.19) | -0.05 (-0.13, 0.03) | 0.05 (-0.04, 0.15) | -0.06 (-0.18, 0.06) | -0.21 (-0.34, -0.07)** |

**Supplementary Table S5.** Generalized estimating equations (GEE) models for comparing the primary and secondary outcomes across study time points between the control and intervention groups with adjustment for age and professional qualification. (^#^ = with adjustment for age and professional qualification; only the model estimates of regression coefficients of the dummy variables for the group [Group: 0= Control (reference); 1= Intervention]; T0=at baseline; T1=immediately after training; T2=3 months after training; Group*T1 and Group*T2=time points and group interaction terms; BRS=Brief Resilience Scale; UWES-9=The 9-item Utrecht Working Engagement Scale short version; ATS-12=The 12-item Anticipated Turnover Scale; SPE=Self-perceived Employability Scale; IWPQ=Individual Work Performance Questionnaire; B=unstandardized coefficient; **P* < .05; ***P* < .01; ****P* < .001).

|  | **n (%)** |
| --- | --- |
| Quality of training received |  |
| Excellent | 28 (17.4%) |
| Good | 100 (62.1%) |
| Fair | 32 (19.9%) |
| Poor | 1 (0.6%) |
| Training matched your anticipation |  |
| Not, definitely not | 3 (1.9%) |
| Not, not really | 23 (14.3%) |
| Yes, generally | 116 (72.0%) |
| Yes, definitely | 19 (11.8%) |
| Training met your needs |  |
| Almost all of my needs | 14 (8.7%) |
| Most of my needs | 90 (55.9%) |
| Only a few of my needs | 54 (33.5%) |
| None of my needs | 3 (1.9%) |
| Recommend the training to friends |  |
| Not, definitely not | 1 (0.6%) |
| No, I don’t think so | 14 (8.7%) |
| Yes, I think so | 116 (72.0%) |
| Yes, definitely | 30 (18.6%) |
| Satisfaction with the amount of help received |  |
| Quite satisfied | 20 (12.4%) |
| Indifferent or mildly satisfied | 42 (26.1%) |
| Mostly satisfied | 86 (53.4%) |
| Very satisfied | 13 (8.1%) |
| The training received helped you to deal with problems more effectively |  |
| Yes, a great deal | 19 (11.8%) |
| Yes, somewhat | 122 (75.8%) |
| No, really don’t help | 20 (12.4%) |
| No, seemed to make things worse | 0 (0.0%) |
| Overall satisfaction with the training received |  |
| Very satisfied | 16 (9.9%) |
| Mostly satisfied | 100 (62.1%) |
| Indifferent or mildly satisfied | 35 (21.7%) |
| Quite satisfied | 10 (6.2%) |
| If you were to seek help again, would you come back to the training |  |
| No definitely | 2 (1.2%) |
| No, I don’t think so | 32 (19.9%) |
| Yes, I think so | 106 (65.8%) |
| Yes, definitely | 21 (13.0%) |

**Supplementary Table S6.** Satisfaction with training among participants in the intervention group (N=161^*^; ^*^There were 32 participants in the intervention group dropped out from the study and had not responded to the training evaluation survey).


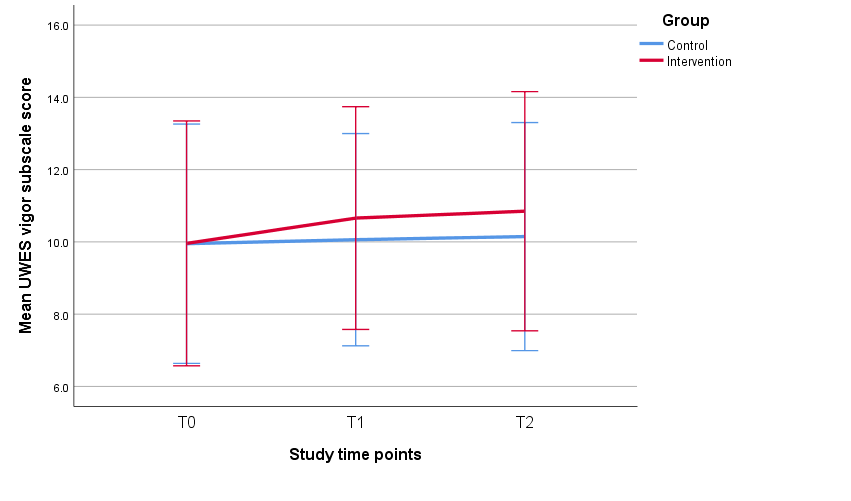

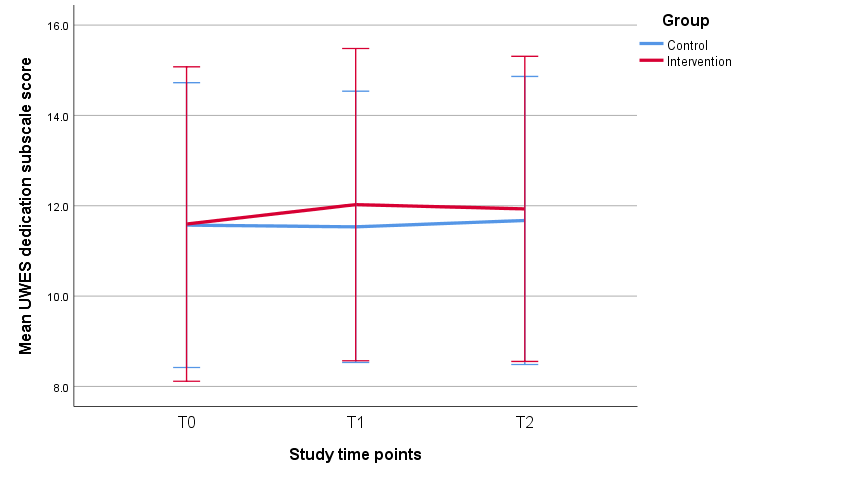


**
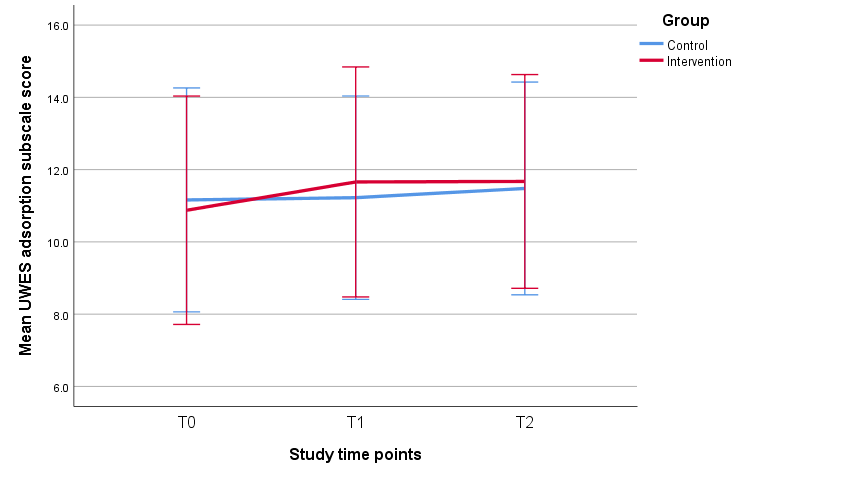

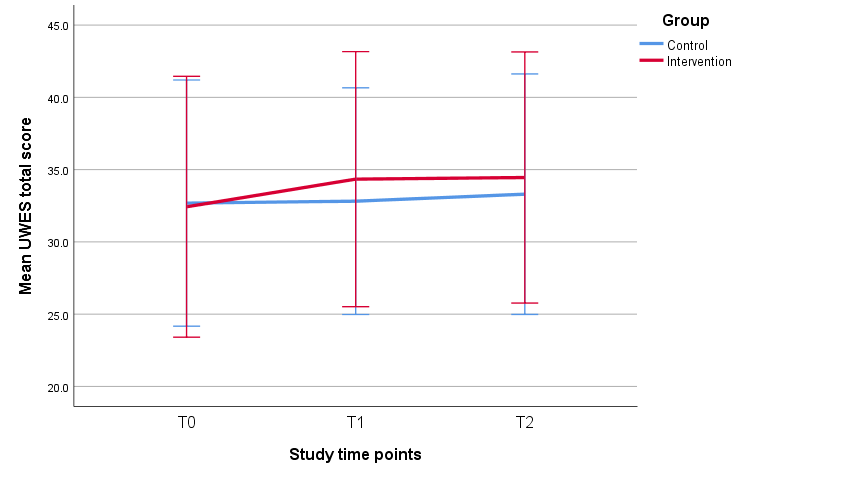
**

**Supplementary Figure S1.** Line charts for short version Utrecht working engagement scale (UWES) showing the means and ± 1 standard deviations of the subscales and total scores across the study time points.


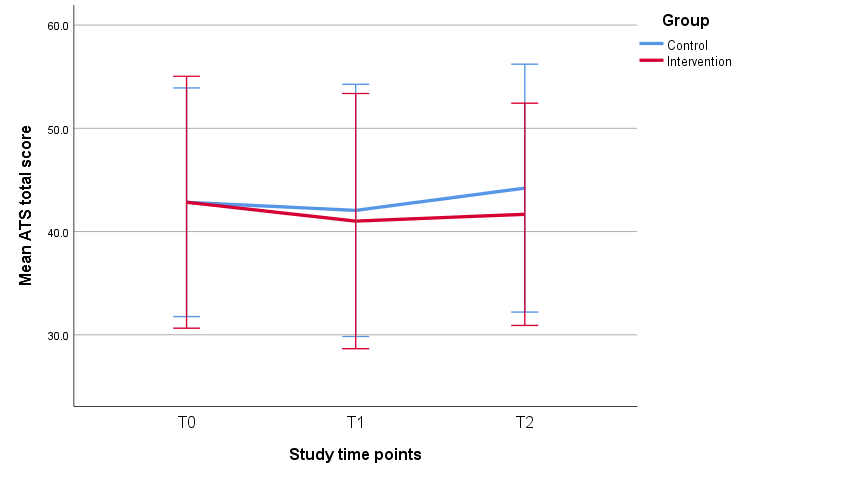


**Supplementary Figure S2.** Line charts for anticipated turnover scale (ATS) showing the means and ± 1 standard deviations of the total scores across the study time points.


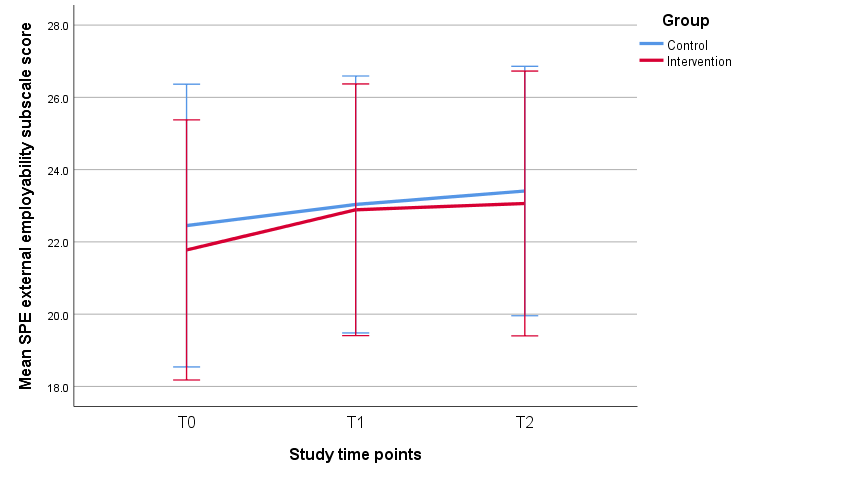

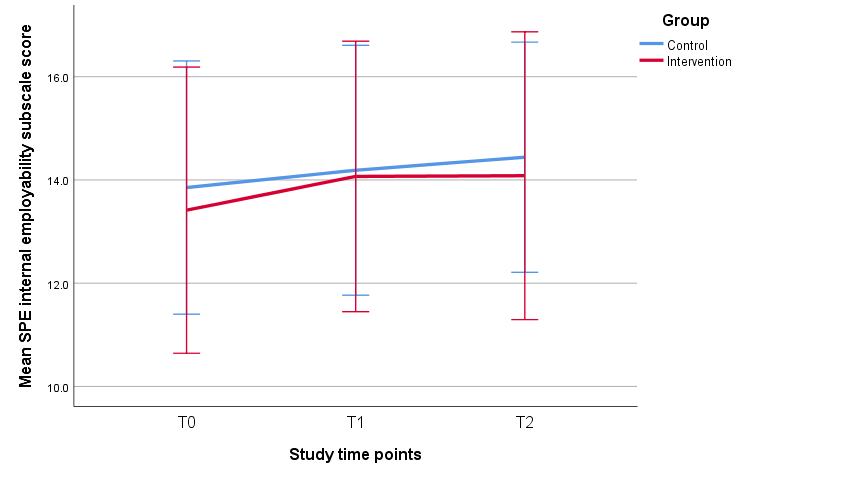

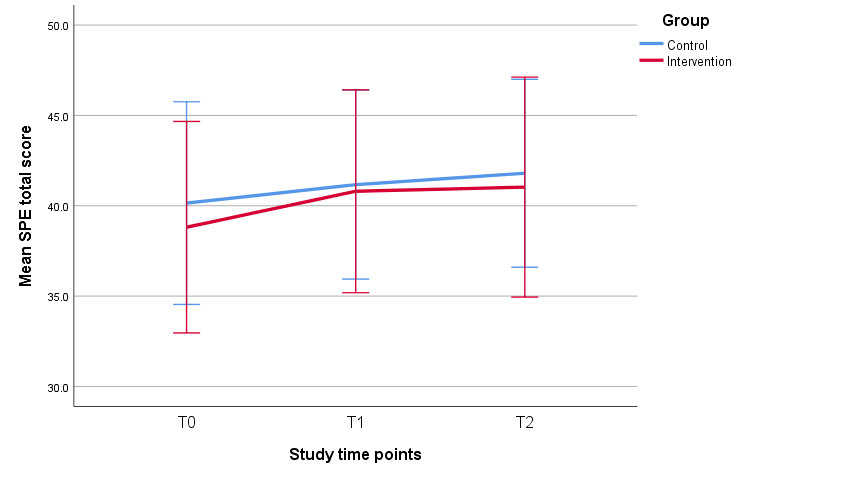


**Supplementary Figure S3.** Line charts for self-perceived employability scale (SPE) showing the means and ± 1 standard deviations of the subscale and total scores across the study time points.

**
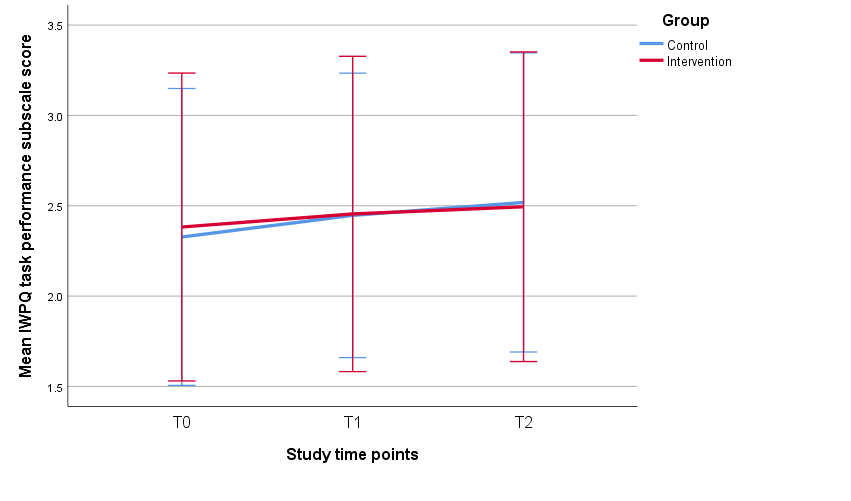

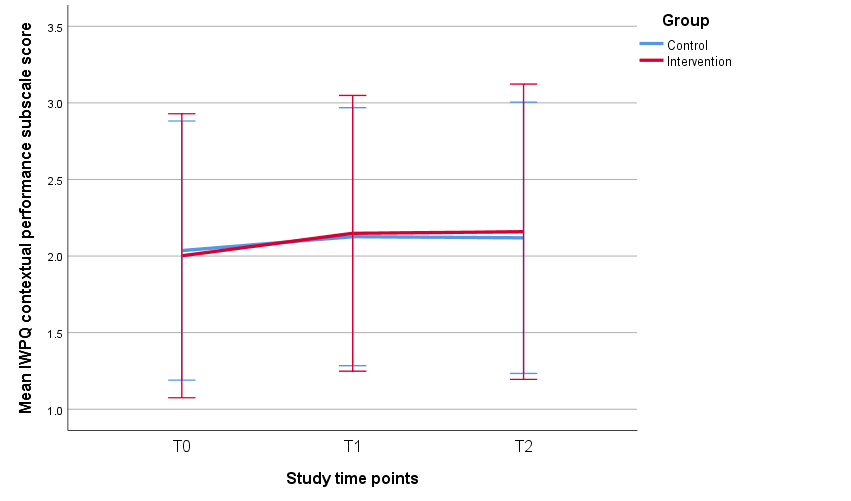

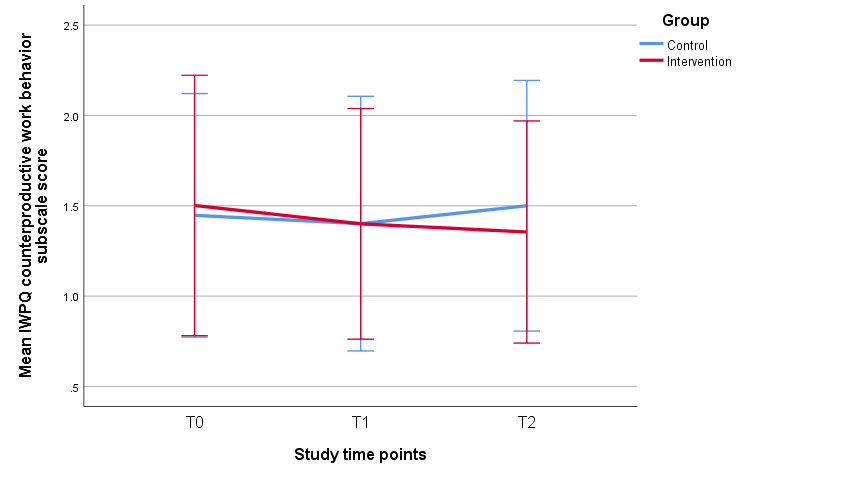
**

**Supplementary Figure S4.** Line charts for individual work performance questionnaire (IWPQ) showing the means and ± 1 standard deviations of the subscale scores across the study time points.
